# A comparison of numerical approaches for statistical inference with stochastic models

**DOI:** 10.1007/s00477-023-02434-z

**Published:** 2023-04-13

**Authors:** Marco Bacci, Jonas Sukys, Peter Reichert, Simone Ulzega, Carlo Albert

**Affiliations:** 1 SIAM, Eawag: Swiss Federal Institute of Aquatic Science and Technology, 8600 Dübendorf, Switzerland; 2 Institute of Computational Life Sciences, ZHAW Zurich University of Applied Sciences, 8820 Wädenswil, Switzerland

**Keywords:** Uncertainty quantification, Bayesian inference, Stochastic model, Hamiltonian Monte Carlo, Particle Markov Chain Monte Carlo, Gibbs sampling, Calibration

## Abstract

Due to our limited knowledge about complex environmental systems, our predictions of their behavior under different scenarios or decision alternatives are subject to considerable uncertainty. As this uncertainty can often be relevant for societal decisions, the consideration, quantification and communication of it is very important. Due to internal stochasticity, often poorly known influence factors, and only partly known mechanisms, in many cases, a stochastic model is needed to get an adequate description of uncertainty. As this implies the need to infer constant parameters, as well as the time-course of stochastic model states, a very high-dimensional inference problem for model calibration has to be solved. This is very challenging from a methodological and a numerical perspective. To illustrate aspects of this problem and show options to successfully tackle it, we compare three numerical approaches: Hamiltonian Monte Carlo, Particle Markov Chain Monte Carlo, and Conditional Ornstein-Uhlenbeck Sampling. As a case study, we select the analysis of hydrological data with a stochastic hydrological model. We conclude that the performance of the investigated techniques is comparable for the analyzed system, and that also generality and practical considerations may be taken into account to guide the choice of which technique is more appropriate for a particular application.

**Supplementary Information:**

The online version contains supplementary material available at 10.1007/s00477-023-02434-z.

## Introduction

Scientists are interested in understanding the function and behavior of environmental systems, and society needs the prediction of their future behavior under given boundary conditions, such as climate change and policy measures, for decision support (Matthies et al. 2007; Reichert et al. 2015; Walling and Vaneeckhaute 2020). However, due to our limited knowledge about the mechanics of complex environmental systems, about their driving forces, and about their intrinsic stochasticity, such predictions are subject to considerable uncertainty (Refsgaard et al. 2007; Beven 2018). This uncertainty combines epistemic uncertainty, due to imperfect knowledge, and aleatory uncertainty, due to inherent variability or stochasticity. Both kinds of uncertainty can be described by probabilities, as they finally lead to a lack of knowledge of the outcome (Reichert et al. 2015; Halpern 2017). The formulation of epistemic probabilities, as well as of mechanisms and aleatory stochasticity, should be inter-subjective (Gillies 1991), e.g., should be elicited from a group of experts or should pass a peer review of experts in the field (Krueger et al. 2012). An established approach to model uncertainty is to describe the intrinsic variability of a system by making the associated mechanistic model stochastic (Soize 2017; Reichert 2020). However, this leads to internal, unknown random variables that may be difficult to infer from data that correspond to output variables of the model (Hartig et al. 2011; Cranmer et al. 2020). This is particularly true for dynamical models, as time-dependent stochastic internal states or parameters lead to a very high-dimensional inference problem that is difficult to solve with numerical algorithms (Reichert et al. 2021; Bacci et al. 2022). It is the overarching goal of this paper to demonstrate and compare some techniques that can be used to numerically implement Bayesian inference for dynamical, stochastic models, and to discuss their advantages and disadvantages and how those depend on the specific modelling setup. To do so, we build on the data and model of a previous study, in which a stochastic process was used to describe the rainfall dynamics in an urban hydrological catchment (Del Giudice et al. 2016) (for clarity and to favor comparisons, we maintain the nomenclature used therein). Indeed, making selected model parameters or inputs stochastic, time-dependent processes, is a technique to better consider intrinsic uncertainty in hydrological models while still exactly maintaining mass balances (Buser 2003; Tomassini et al. 2009; Reichert and Mieleitner 2009; Reichert et al. 2021; Bacci et al. 2022). The approach of making parameters stochastic in addition to stochastic mass-balance equations has also been suggested (Liu and West 2001; Liu and Gupta 2007; Suweis et al. 2010), but that allows fluctuations in mass-balances and not just in mass fluxes, which violate mass conservation.

A prominent motivation to borrow our case study from the hydrological literature, aside from our own familiarity with it, is that hydrological systems are often described with deterministic process models, where the effect of all uncertainties on the output is accounted for by just one additive lumped error term, potentially on a transformed scale, and potentially accounting for autocorrelation empirically (Sorooshian and Dracup 1980; Kuczera 1983; Bates and Campbell 2001; Schoups and Vrugt 2010; Ammann et al. 2019). While this approach is computationally efficient, it is not fully satisfying conceptually, as it does only incompletely consider the uncertainty in internal model states, and needs to impose the autocorrelation generated by the internal mechanisms through an empirical parameterization (Blöschl and Sivaplalan 1995; Kuczera et al. 2006; Reichert et al. 2021).

There may be three main reasons why a large fraction of hydrological modelling studies are still based on deterministic hydrological process models. First, many hydrologists are familiar with this framework and may not see the benefit of expanding it. Second, statistical inference with stochastic process models is algorithmically and numerically much more demanding than when using deterministic models with an additive stochastic error term. Third, it is often not straightforward to choose a numerical technique and find an implementation easily adaptable to a specific hydrological problem of interest. Hence, by referring to a hydrological case study, we take the opportunity to enrich the discussion by leveraging on all these specific application domain aspects.

Accordingly, we first describe three numerical techniques that are well suited to perform Bayesian inference with hydrological models with stochastic inputs and/or parameters. Second, we conduct a comparative analysis of the results and sampling efficiency for all of these techniques, analyzing under which circumstances which technique may be most useful. Third, we provide the implementation of all the methods as open source software to facilitate their practical application, although the referenced software tools stand at different levels of simplicity of reuse and of numerical optimization. Thus, we refrain from comparing pure computational requirements and performances, although this aspect is qualitatively discussed.

More specifically, the paper is structured as follows. In Sect. 2 we review the general theory, and the three numerical techniques that we use for calibration of the selected case study, which are Hamiltonian Monte Carlo  (Duane et al. 1987; Neal 2011), Particle Markov Chain Monte Carlo (Andrieu et al. 2010), and Conditional Ornstein-Uhlenbeck Sampling (Buser 2003; Tomassini et al. 2009). Consequently, in Sect. 3, we illustrate the case study, that is, the hydrological system, the available data, and the modeling choices that we make. Section 4 is devoted to the application of the numerical techniques to the calibration of the chosen stochastic hydrological model to the available data, and to expose the relevant comparisons. Finally, in Sect. 5, we conclude with a summary of the suitability of the different methods, recommendations, and suggestions for further research.

## Methods

### Stochastic models

The inference methods compared in this paper apply to dynamic, stochastic process models that: (i) have one or multiple unobserved internal states $\varvec{\xi }(t)$ that are modelled as a (potentially multi-dimensional) random process; (ii) present a set of observable variables ${\textbf{y}}$. Our model is then defined by the joint probability density of the random process $\varvec{\xi }$ and model parameters $\pmb {\theta }$,

1a $$\begin{aligned} f_{{\rm M}}(\varvec{\xi },\pmb {\theta }) = f_{\varvec{\Xi }} (\varvec{\xi } \vert \pmb {\theta }) \cdot f_{\varvec{\Theta }}(\pmb {\theta }), \end{aligned}$$

together with a function, ${\textbf{y}}_{{\rm M}}$, that describes how the observable variables ${\textbf{y}}$ depend on the internal state and parameters:

1b $$\begin{aligned} {\textbf{y}} = {\textbf{y}}_{{\rm M}}(\varvec{\xi },\pmb {\theta }). \end{aligned}$$

In these equations, $f_{\varvec{\Xi }}$ is the probability density function of the random process given the parameters and $f_{\varvec{\Theta }}$ is the probability density function of the parameters. The probability density of ${\textbf{y}}$, obtained by propagating the probability density $f_{{\rm M}}$ through the function ${\textbf{y}}_{{\rm M}}$, describes our knowledge about the output of the process model.

To be able to generate realistic synthetic observations, or to use real data to calibrate model parameters and infer model states, we need to be able to express the probability of obtaining any observable value ${\textbf{y}}_{\rm o}$ for given model output ${\textbf{y}}$, state $\varvec{\xi }$, and parameters $\varvec{\theta }$. In sufficiently general terms, this can be expressed with an observational model such as:

2 $$\begin{aligned} f_{{\textbf{Y}}_{\rm o}}({\textbf{y}}_{{\rm o}} \mid {\textbf{y}},\varvec{\xi },\varvec{\theta }). \end{aligned}$$

The explicit definition of the observational model depends on the application. For instance, particular cases of (2) are (3a) and (3b) as follows. If the intended purpose of (2) is to model the observational error of the measurement device, then it is typically formulated in the form:

3a $$\begin{aligned} f_{{\textbf{Y}}_{\rm o}}({\textbf{y}}_{{\rm o}} \mid {\textbf{y}},\varvec{\xi },\varvec{\theta }) = f_{{\textbf{Y}}_{\rm o}}^\mathrm {(a)}({\textbf{y}}_{{\rm o}} \mid {\textbf{y}}, \varvec{\theta }). \end{aligned}$$

If instead the goal is to quantify how the uncertainty of the internal state of the process model manifests on the observable variables, then a model of the type:

3b $$\begin{aligned} f_{{\textbf{Y}}_{\rm o}}({\textbf{y}}_{{\rm o}} \mid {\textbf{y}},\varvec{\xi },\varvec{\theta }) = f_{{\textbf{Y}}_{\rm o}}^\mathrm {(b)}({\textbf{y}}_{{\rm o}} \mid \varvec{\xi }, \varvec{\theta }), \end{aligned}$$

can be more appropriate. Combinations of (3a) and (3b) are possible when the observable variables are, e.g., multiple and independent, ${\textbf{y}}_{{\rm o}} = [{\textbf{y}}^{(a)}_{{\rm o}},\, {\textbf{y}}^{(b)}_{{\rm o}}]$:

3c $$\begin{aligned} f_{{\textbf{Y}}_{\rm o}}({\textbf{y}}_{{\rm o}} \mid {\textbf{y}},\varvec{\xi },\varvec{\theta }) = f_{{\textbf{Y}}_{\rm o}}^\mathrm {(a)}({\textbf{y}}^{(a)}_{{\rm o}} \mid {\textbf{y}}, \varvec{\theta }) f_{{\textbf{Y}}_{\rm o}}^\mathrm {(b)}({\textbf{y}}^{(b)}_{{\rm o}} \mid \varvec{\xi }, \varvec{\theta }). \end{aligned}$$

We finally note that, to simplify the notation, in all our equations the parameter vector $\pmb {\theta }$ combines the parameters of the system and observational models, as well as those of the random process.

### Bayesian inference

Bayesian inference provides us with the probabilistic framework to infer both the time course of the stochastic process $\varvec{\xi }(t)$ and the values of the constant parameters $\pmb {\theta }$. By using the definitions of the model structure given in Sect. 2.1, we can formulate the joint probability density of observations, states and model parameters:

4 $$\begin{aligned} f_{{\textbf{Y}}_{\rm o},\varvec{\Xi },\varvec{\Theta }}( {\textbf{y}}_{{\rm o}}, \varvec{\xi },\varvec{\theta } ) = f_{{\textbf{Y}}_{\rm o}}( {\textbf{y}}_{{\rm o}} \mid {\textbf{y}}_{{\rm M}}(\varvec{\xi },\pmb {\theta }), \varvec{\xi }, \varvec{\theta } ) \cdot f_{{\rm M}}(\varvec{\xi },\pmb {\theta }). \end{aligned}$$

Given specific observations ${\textbf{y}}_{\rm o} = {\textbf{y}}_{{\rm obs}}$, Bayes’ rule reads from (4) and (1) as:

5 $$\begin{aligned} \begin{aligned} f_{{\rm post}}( \varvec{\xi },\pmb {\theta }\mid \textbf{y}_{{\rm obs}}) \propto&\, f_{{\textbf{Y}}_{\rm o},\varvec{\Xi },\varvec{\Theta }}( {\textbf{y}}_{{\rm obs}}, \varvec{\xi },\varvec{\theta } ) \\ =&\, f_{{\textbf{Y}}_{\rm o}} \bigl ( {\textbf{y}}_{{\rm obs}} \mid {\textbf{y}}_{{\rm M}}(\varvec{\xi },\pmb {\theta }), \varvec{\xi },\varvec{\theta } \bigr ) \, \cdot \\&\quad f_{\varvec{\Xi }} (\varvec{\xi } \mid \pmb {\theta }) \cdot f_{\varvec{\Theta }}(\pmb {\theta }). \end{aligned} \end{aligned}$$

The probability distribution of ${\textbf{y}}$ is again given by propagating this posterior distribution through the function ${\textbf{y}}_M$ given by Eq. (1b). In equation 5, probability densities $f_{\varvec{\Xi }} (\varvec{\xi } \vert \pmb {\theta })$ and $f_{\varvec{\Theta }}(\pmb {\theta })$ are customarily called prior densities, while those distributions that use the observed data such as $f_{{{\bf Y}}_{{\rm o}}} \bigl ( {\textbf{y}}_{{\rm obs}} \vert {\textbf{y}}, \varvec{\xi },\varvec{\theta } \bigr )$ define the likelihood. Of note, in Sect. 3 we decompose the likelihood for our hydrological case study into two independent parts, one for the river discharge and the other for the rainfall. Therefore, the explicit form of (5) that we use is based on (3c):

6 $$\begin{aligned} \begin{aligned} f_{{\rm post}}(\varvec{\xi },\pmb {\theta }\mid \textbf{y}_{{\rm obs}}) \propto \,&f_{{\textbf{Y}}_{\rm o}}^\mathrm {(a)}( {\textbf{y}}^{(a)}_{{\rm obs}} \mid {\textbf{y}}_{{\rm M}}(\varvec{\xi },\pmb {\theta }), \varvec{\theta }) \cdot \\&\quad f_{{\textbf{Y}}_{\rm o}}^\mathrm {(b)}( {\textbf{y}}^{(b)}_{{\rm obs}} \mid \varvec{\xi }, \varvec{\theta }) \cdot f_{{\rm M}}(\varvec{\xi },\pmb {\theta }) \\ = \,&f_{{\textbf{Y}}_{\rm o}}^\mathrm {(a)}( {\textbf{y}}^{(a)}_{{\rm obs}} \mid {\textbf{y}}_{{\rm M}}(\varvec{\xi },\pmb {\theta }), \varvec{\theta }) \cdot f_{{\textbf{Y}}_{\rm o}}^\mathrm {(b)}( {\textbf{y}}^{(b)}_{{\rm obs}} \mid \varvec{\xi }, \varvec{\theta }) \cdot \\&\quad f_{\varvec{\Xi }} (\varvec{\xi } \mid \pmb {\theta }) \cdot f_{\varvec{\Theta }}(\pmb {\theta }), \end{aligned} \end{aligned}$$

with ${\textbf{y}}^{(a)}_{{\rm obs}}$ and ${\textbf{y}}^{(b)}_{{\rm obs}}$ being the discharge and rainfall data of our specific application, respectively, and $f_{{\textbf{Y}}_{\rm o}}^\mathrm {(a)}$ and $f_{{\textbf{Y}}_{\rm o}}^\mathrm {(b)}$ the respective observational models, see Sects. 3.2.1 and 3.2.2. Importantly, the fact that $f_{{\textbf{Y}}_{\rm o}}^\mathrm {(b)}$ does not explicitly use the ${\textbf{y}}^{(b)}$ component of the model output (rainfall) does not limit our ability to infer ${\textbf{y}}^{(b)}$ thanks to relationship (1b), which is made explicit by equation (9) in Sect. 3.1. Model structures of the form (1) and (2), with corresponding inference according to (5), appear in many applications, e.g., for models with stochastic, time-dependent parameters Liu and Gupta (2007); Tomassini et al. (2009); Reichert and Mieleitner (2009); Leisenring and Moradkhani (2010); Reichert et al. (2021); Bacci et al. (2022).

### Numerical approaches to bayesian inference for stochastic models

The very high-dimensionality of the posterior (5), resulting from the time series $\varvec{\xi }$, makes Bayesian inference with standard numerical algorithms, such as the Metropolis algorithm, very inefficient and thus for practical reasons (too slow convergence) not applicable.

If forward simulations from the model are fast and we are only interested in the marginal posterior for the parameters $\pmb {\theta }$, we may resort to Approximate Bayesian Computation (e.g. Beaumont et al. (2002); Lenormand et al. (2013); Albert et al. (2015)), which is based on comparing simulations with measurements in terms of a small set of summary statistics that retain most of the $\pmb {\theta }$-related information in $\textbf{y}_{{\rm obs}}$. However, in many cases we are also interested in the posterior of the stochastic process $\varvec{\xi }$. For instance, if $\varvec{\xi }$ denotes a time-dependent parameter, its inferred time-course can give us clues about how to improve the model (Reichert and Mieleitner 2009; Bacci et al. 2022). In our case study in Sect. 3, we are interested in inferring the real rainfall from both rain and runoff observations. In such cases, we have to solve a very high-dimensional inference problem, jointly for $\pmb {\theta }$ and $\varvec{\xi }$. Hence, ABC would be too inefficient.

In what follows in this contribution, we restrict our consideration to *sampling* techniques of the posterior. As mentioned above, plain random-walk based sampling algorithms are grossly inefficient in high-dimensional spaces due to extremely low acceptance rates. Hence, we compare three different remedies for this problem. Hamiltonian Monte Carlo employs Hamiltonian dynamics to achieve high acceptance rates even for large step sizes in high-dimensional sampling spaces. Particle filters iterate piece-wise forward simulations of the stochastic model with observation-based importance sampling, thus constraining the sampling of the high-dimensional process $\varvec{\xi }$ by the data. Finally, Gibbs-sampling can be used to increase the acceptance rate by sampling only a subset of all the variables at a time. If the stochastic process that is used in the model allows for bridge-sampling, i.e. sampling sub-intervals of the time-interval with fixed end-points, Gibbs sampling can be very efficient. This is the case for the Ornstein-Uhlenbeck process that we use in our case study. In the remainder of this section, we describe the three different techniques in more detail.

#### Hamiltonian Monte Carlo (HMC)

HMC implements a dynamical system that integrates Hamilton’s equations in some auxiliary time (not to be confused with the time of the model) using a potential energy surface given by the negative logarithm of the posterior density. This ensures to obtain unbiased samples from the posterior, and hence that the acceptance probability would be 1 in the absence of discretization errors. In practice, however, it is not 1 due to the necessary discretization of the auxiliary time, which leads to an error that is corrected by means of a Metropolis accept/reject step at the end of each time integration of the system. Still, HMC is much more efficient than random walk type Metropolis algorithms (Duane et al. 1987; Neal 2011), and allows sampling even very high dimensional spaces, although it requires calculating the *gradient* of the target distribution. In its simplest variant, the Hamiltonian reads as $H(\varvec{\xi },\pmb {\theta };\varvec{\zeta },\varvec{\pi })=-\ln f_{{\rm post}}(\varvec{\xi },\pmb {\theta }\mid \textbf{y}_{{\rm obs}}) +\sum _{i}\zeta _i^2/(2m_i)+\sum _{\alpha }\pi _\alpha ^2/(2M_\alpha )$, where the $\varvec{\zeta }$ and $\varvec{\pi }$ are auxiliary degrees of freedom interpreted as momenta conjugate to the configurational variables $\varvec{\xi }$ and $\pmb {\theta }$, respectively.^1^ The masses $m_i$ and $M_\alpha$ are tuning parameters of the algorithm. Their choice can partly be automatized. HMC generates samples from $\exp [-H(\varvec{\xi },\pmb {\theta };\varvec{\zeta },\varvec{\pi })]$, the marginalization of which w.r.t. the momenta constitutes a sample from the posterior. Each update step consist of a random draw of the momenta (from a Gaussian), followed by an integration of Hamilton’s equations in the auxiliary time. The sampling of the momenta at the beginning of each step makes sure that all energy shells are sampled.

HMC allows for a high acceptance rate and low auto-correlation, at the computational cost of a fine discretization and a long integration time, respectively. For automatically finding the integration time interval, the so-called No-U-Turn Sampler (NUTS) can be employed (Hoffman and Gelman 2014). The tuning of the masses should be driven by adapting the mass matrix to the curvature of the energy landscape. A natural choice is the inverse of the *Fisher metric*, which is however position-dependent and renders the implementation significantly more difficult (Girolami and Calderhead 2011). A simpler variant of this idea of enhancing HMC by means of Riemann geometry recently appeared in Hartmann et al. (2022).

The Hamiltonian dynamics that emerges from problems of the kind considered here typically happens on very different time-scales (again referring to the auxiliary time), associated with the typically large and potentially very different numbers of measurement time points on the one hand, and discretization time points needed for the stochastic process $\xi$ on the other hand. Therefore, in this work, we employ a time-scale separation based on Trotter’s formula (Albert et al. 2016). We tune masses and integration time manually.

#### Particle Markov Chain Monte Carlo (PMCMC)

States of state-space or hidden Markov Models are often inferred using the Ensemble Kalman Filter (EKF) (Evensen 2009). This approach has the advantage of being very efficient, but the disadvantage of relying on linear approximations the accuracy of which is difficult to assess.

Alternative approaches are particle filters or particle smoothers, which filter the distribution of the states of the model according to the data. An ensemble of model realizations, called “particles”, is propagated through the observations combining model propagation from one observation to the next with resampling of the model states using as weights the likelihood of the respective observation at each time point (Künsch 2001; Godsill et al. 2004; Fearnhead and Künsch 2018; Van Leeuwen et al. 2019). The difference between particle filters and smoothers is that the former condition each state on current and past observations, while the latter condition on the full time series, including future observations. Particle Markov Chain Monte Carlo techniques combine particle filtering or smoothing for the states with Markov Chain Monte Carlo (MCMC) for the constant parameters, either based on an approximation to the marginal likelihood calculated from the particle ensemble at each step of the Markov chain, or by Gibbs sampling between states and parameters (Andrieu and Roberts 2009; Andrieu et al. 2010; Kantas et al. 2015; Kattwinkel and Reichert 2017; Sukys and Kattwinkel 2018).

#### Conditional Ornstein-Uhlenbeck sampling (COUS)

By using a potentially transformed Ornstein-Uhlenbeck process to describe input or intrinsic stochasticity in the model, we can profit from its structure. To reduce the rejection rate when proposing a new realization of this process, instead of proposing a new realization of the full time series, we first divide the time domain into a random set of sub-intervals. We then sequentially re-sample the Ornstein-Uhlenbeck process just within one sub-interval at a time, conditional on the values at the end points of the interval to guarantee continuity of the process. The rejection rate can then be adapted by the selection of the number of intervals. Using more (shorter) intervals decreases the rejection rate at the expense of more simulations to be done for covering the whole time domain. We combine this sub-sampling strategy for the random process with Metropolis or Metropolis-Hastings sampling of the constant parameters in an overarching Gibbs sampling framework (Buser 2003; Tomassini et al. 2009; Reichert and Mieleitner 2009; Reichert et al. 2021; Bacci et al. 2022). Figure 2 in Reichert et al. (2021) illustrates the conditional proposals in these sub-intervals and their acceptance or rejection. For more details, see the original publications (Buser 2003; Tomassini et al. 2009).

### Implementation

HMC is implemented from scratch in C++14 using the open-source ADEPT library (Automatic Differentiation using Expression Templates, version 1.1) to calculate the gradient of the Hamiltonian (Hogan 2014). The automatic differentiation feature, in particular, allows us to automatize the HMC algorithm to a large extent thus making the HMC approach very general and suitable for a broad range of applications. Indeed, only the Hamiltonian needs to be modified according to the specific case study, while the implementation of the algorithm remains essentially unaltered.

All the modeling components required to perform PMCMC for the chosen hydrological system are built within the inference framework SPUX (Sukys and Kattwinkel 2018; Sukys and Bacci 2021). SPUX is a modular framework for Bayesian inference written in Python. It aims to enable uncertainty quantification for stochastic models across different disciplines. Indeed, SPUX is not tailored towards a specific application domain, and can be coupled to models written in different programming languages. It is a hub for different numerical techniques, including PMCMC and SABC (Albert et al. 2015), and can easily leverage distributed memory systems for parallelization.

COUS is implemented in the R package timedeppar (https://cran.r-project.org/package=timedeppar) (Reichert et al. 2021; Reichert 2022). The differential equations of the hydrological model are integrated using the R package deSolve http://cran.r-project.org/package=deSolve with an implementation of the right-hand side of the differential equations in C to improve efficiency. The short time series of our case study allow us to integrate the whole time series to evaluate the likelihood once part of the stochastic input has been modified as described in Sect. 2.3.3. This makes the interface to the simulation program particularly simple as it just has to be able to process time series of inputs or parameters. However, this procedure would scale poorly with the length of the time series. The package timedeppar (https://cran.r-roject.org/package=timedeppar) thus allows the user to store the internal state from the previous simulation and only re-calculate and replace the part of the time series that is (strongly) affected by the modified input. For differential equation models as used in our case study, this part starts with the start point of the modification, and ends after some characteristic times of the slowest time scale of the model after the end point of the modification. The code published with (Reichert et al. 2021) demonstrates how this can be done.

### Comparing efficiency

As the different numerical approaches are implemented on different computing platforms, required CPU time is not useful to compare the numerical effort. Similarly, we cannot directly compare the chain lengths of the Markov chains as the auto-correlation is different. To reduce bias in our metrics, we thus calculate the ratio of the obtained effective sample size and the number of posterior evaluations, called “function calls”. This is still not completely unbiased, as HMC requires not just the evaluation of the posterior, but also its gradient, and PMCMC, through its iterative integration, has more overhead compared to COUS or HMC which integrate the full time series at once.

Numerically, the effective sample size is calculated using the implementation in the R (R Core Team 2020) software package mcmcse (https://cran.r-project.org/package=mcmcse) for both uni- and multi-variate cases (Vats et al. 2019). The number of function calls $N_{{\rm fc}}$ that are required to obtain the posterior samples for the three techniques, HMC, PMCMC, and COUS are given by

7a $$\begin{aligned} N^{\text{PMCMC}}_{{\rm fc}}&= N_{{\rm MC}} \times N_{{\rm p}} \end{aligned}$$

7b $$\begin{aligned} N^{\text{COUS}}_{{\text fc}}&= N_{{\rm MC}} \times (N_{{\rm i}} +1) \end{aligned}$$

7c $$\begin{aligned} N^{\text{HMC}}_{{\text fc}}&= N_{{\rm MC}} (1+L), \end{aligned}$$

where $N_{{\rm MC}}$ is the length of the Markov chain, $N_{\rm p}$ is the number of particles in PMCMC, $N_{\rm i}$ is the number of sub-intervals in COUS, and *L* is the number of gradient-evaluations needed in HMC to obtain a sample point per energy evaluation. Here, we use $L=6$ discretization steps for Hamilton’s equations, and assume that gradient- and energy-evaluations cost approximately the same.

## Case study

Our case study is based on one of the events used for describing sewer discharge under consideration of stochastic rain input by Del Giudice et al. (2016). As we focus on a single, short event, we can simplify the original model by omitting the daily variation in the flow that was used to model the dynamics of the outflow of the sewage treatment plant. This setting is then well suited to compare numerical approaches to Bayesian inference of states and model parameters under the difficulty of inferring a stochastic process (here rainfall), but still with tractable computational requirements due to the short duration of the event.

### Hydrological model

The simplified model is given by the following mass-balance differential equation for the water stored in the sewer:

8 $$\begin{aligned} \frac{\text{d}S(t)}{\text{d}t} = A P(t) + Q_{\rm gw} - Q(t) \quad \text{ with } \quad Q(t) = \frac{S(t)}{K} \quad , \end{aligned}$$

where *t* is time, *S* is water volume in the sewer, *A* is catchment area, *P* is precipitation, $Q_{\rm gw}$ is constant infiltration of groundwater, *Q* is discharge, and *K* is the mean residence time of the water in the reservoir (see also Table 1 for a list of all variables and their units).

**Table 1:** Variables, units, and descriptions. From top of the table, the first group of variables are the input, state and parameters of the hydrological model. The second group are the parameters of the rainfall and rain observation models. The group at the bottom encompasses the parameters that are fixed to values taken from Del Giudice et al. (2016)

Variable	Unit	Description
$\xi$		Rainfall potential
P	l/(s m$^2$)	Rainfall rate
S	l	Water volume in the reservoir
Q	l/s	Runoff rate (discharge)
K	s	Reservoir retention time
$Q_{\rm gw}$	l/s	Groundwater base flow
*W*	–	Wiener process (Brownian motion)
$\sigma _z$	l/s	Standard deviation of the discharge observation model
$\sigma _\xi$		Standard deviation of the rainfall obs. model on the $\xi$ scale
$\uplambda$	l/(s m$^2$)	Scaling factor within rainfall transformation *r*
$\gamma$	–	Exponential factor within rainfall transformation *r*
$\xi _r$		Domain split location within rainfall transformation *r*
$\alpha$	l/s	Coefficient of the discharge observation model (25)
$\beta$	l/s	Coefficient of the discharge observation model (50)
$\tau$	s	Autocorrelation time of the OU process (636 s)
A	m$^2$	Catchment area (11815.8 m$^2$)

The rainfall *P* is given by a transformation of the rainfall potential, $\xi$, (Sigrist et al. 2012; Del Giudice et al. 2016):

9 $$\begin{aligned} P(t) = r \bigl (\xi (t)\bigr ) = \left\{ \begin{array}{cl} \uplambda \bigl (\xi (t) - \xi _r\bigr ) ^ {1 + \gamma } &{} \text{ if } \xi (t) > \xi _r \\[1ex] 0 &{} \text{ if } \xi (t) \le \xi _r \end{array} \right. \end{aligned}$$

which is assumed to follow the standardized Ornstein-Uhlenbeck stochastic process (10):

10 $$\begin{aligned} \xi (t) \mid \xi (s) \sim \text{N}\left( \xi (s) \exp \left( -\frac{t-s}{\tau } \right) , \right. \nonumber \\ \left. \; \sqrt{1 - \exp \left( -2\frac{t-s}{\tau } \right) } \right) , \end{aligned}$$

that is, a process that fulfills the stochastic differential Eq. (11):

11 $$\begin{aligned} \text{d}\xi (t) = - \frac{1}{\tau } \xi (t) + \sqrt{\frac{2}{\tau }} \text{d}W(t) \end{aligned}$$

(see Table 1 for the meaning and units of the variables). Stochasticity of rainfall is thus generated by the stochastic Ornstein-Uhlenbeck process, whereas the skewed distribution of rainfall and the finite probability of zero rain are generated by the nonlinear, partly non-invertible transformation *r* given by Eq. (9).

### Observation models

As we are inferring the stochastic process of the rainfall potential as well as model parameters from rainfall and discharge observations, we need observation models for rainfall and discharge (see Eq. 3). Note that both of these “observation models” are partly lumped error models, as they also have to consider any effects of structural weaknesses of our rain and hydrology descriptions. They are only partly lumped, as parametric uncertainty as well as input uncertainty are considered explicitly in addition to the “observation error models”.

#### Stream flow observation model

The model of observed discharge can be formulated as conditional on the predicted discharge in the form of Eq. (3a). It is convenient to formulate a normal and homoscedastic error model on a transformed *z*-scale and get the required skewness and heteroscedasticity by the back-transformation to the discharge scale. We use the transformation applied already in Del Giudice et al. (2016):

12 $$\begin{aligned} z = H(Q) = \beta \log \left( \sinh \left( \frac{\alpha + Q}{\beta }\right) \right) . \end{aligned}$$

The normally distributed error on this scale reads as (with standard deviation $\sigma _z$):

13 $$\begin{aligned} {f}_{Z_{\rm o}}\bigl (H(Q_{\rm o}) \mid Q \bigr ) = f_{{\rm N}( H(Q), \sigma _z )}\bigl (H(Q_{\rm o})\bigr ), \end{aligned}$$

Transformation to discharge then leads to the probability density for observed discharge:

14 $$\begin{aligned} {f}_{Q_{\rm o}}^{(a)}(Q_{\rm o} \mid Q) = f_{{\rm N}( H(Q), \sigma _z )}\bigl (H(Q_{\rm o})\bigr ) \; \frac{\text{d}H}{\text{d}Q} (Q_{\rm o}) \end{aligned}$$

with

$$\begin{aligned} \frac{\text{d}H}{\text{d}Q}(Q_{\rm o}) = \frac{1}{\tanh \bigl ((\alpha + Q_{\rm o}) / \beta \bigr )}. \end{aligned}$$

The above formulation implies that multiple observations are assumed independent, such that the joint likelihood reads:

15 $$\begin{aligned} f_{{\bf Q}_{\rm o}}^{(a)}(\textbf{Q}_{\rm o} \mid \textbf{Q}) = \prod _{i=1}^{N_Q}{ {f}_{Q_{\rm o}}^{(a)}(Q_{\rm o} = Q_{\rm oi} \mid Q = Q_i)}, \end{aligned}$$

where the index *i* indexes the times when there are runoff observations $Q_{\rm oi}$, with $Q_{\rm i}$ being the corresponding model output.

#### Precipitation observation model

The observation model for rainfall, given the rainfall potential $\xi (t)$, is formulated as a normal distribution in the space of the rainfall potential centered at $\xi (t)$ and with standard deviation $\sigma _\xi$, and is denoted $\xi _{\rm o}(t)$. This distribution is then transformed to rainfall observations by the transformation (9): $P_{\rm o}(t)=r\bigl (\xi _{\rm o}(t)\bigr )$. This leads to an observation model in the form of (3b). As $\xi$-values below $\xi _{\rm r}$ are transformed to zero by the transformation (9), the probability of zero observed rain is given as

16 $$\begin{aligned} \begin{aligned} {p}\Bigl (\bigl ( P_{\rm o}(t) \mid \xi (t) \bigr ) = 0 \Bigr ) =&\,\, {p}\Bigl ( \bigl ( \xi _{\rm o}(t) \mid \xi (t) \bigr ) < \xi _{\rm r} \Bigr ) = \\ =&\int _{-\infty }^{\xi _r} f_{{\rm N}(\xi (t),\sigma _\xi )}(\xi ') \text{d}\xi ' = F_{{\rm N}(\xi (t),\sigma _\xi )} (\xi _{\rm r}) \quad , \end{aligned} \end{aligned}$$

where $f_{{\rm N}}$ and $F_{{\rm N}}$ denote, respectively, the probability density and cumulative distribution function of the normal distribution with mean and standard deviation given in the subscript. The probability density of the rainfall potential corresponding to positive rain observations is given as:

17 $$\begin{aligned} {f}_{\Xi _{\rm o}}\bigl (r^{-1}(P_{\rm o}(t)) \mid \xi (t) \bigr ) = f_{{\rm N}(\xi (t),\sigma _\xi )} \bigl (r^{-1}(P_{\rm o}(t))\bigr ) \quad \text{ for } P_{\rm o}(t) > 0 \end{aligned}$$

(note that the transformation *r* according to Eq. (9) is invertible for $P > 0$). Transforming this distribution to the rain observations thus leads to:

18 $$\begin{aligned} {f}_{P_{\rm o}}^{(b)}\bigl (P_{\rm o}(t) \mid \xi (t) \bigr ) = \frac{f_{{\rm N}(\xi (t),\sigma _\xi )} \bigl (r^{-1}(P_{\rm o}(t))\bigr )}{\displaystyle \frac{\text{d}r}{\text{d}\xi }\bigl (r^{-1}(P_{\rm o} (t))\bigr )} \quad \text{ for } P_{\rm o}(t) > 0 \end{aligned}$$

with

$$\begin{aligned}{} & {} \frac{\text{d}r}{\text{d}\xi }(r^{-1}(P_{\rm o}(t))\bigr ) = \uplambda (1+\gamma ) \bigl (r^{-1}(P_{\rm o}(t)) - \xi _r\bigr ) ^ { \gamma } \\{} & {} \quad = \uplambda (1 + \gamma ) \left( \frac{P_{\rm o}(t)}{\uplambda }\right) ^{\frac{\gamma }{1+\gamma }}. \end{aligned}$$

As with the discharge, the joint likelihood is the independent composition of the marginal likelihoods (16) and (18), and writes:

19 $$\begin{aligned}{} & {} f_{{\bf P}_{\rm o}}^{(b)}\bigl (\textbf{P}_{\rm o} \vert{\varvec{\xi }} \bigr ) = \prod _{i=1}^{N_{P=0}}{ {p}\Bigl (\bigl ( P_{\rm o}(t_i) \vert\xi (t_i) \bigr ) = 0 \Bigr )}\nonumber {} \prod _{j=1}^{N_{P>0}}{ {f}_{P_{\rm o}}^{(b)}\bigl (P_{\rm o}(t_j) \vert \xi (t_j) \bigr )}, \end{aligned}$$

where the index *i* runs on the times when there is no rainfall while *j* indexes when rain is observed.

### Priors for inferred parameters

We list in Table 2 the prior assumptions for the parameters of our models. The joint prior is obtained assuming that all the listed marginal distributions are independent.

**Table 2:** Joint prior density is simply the product of univariate densities for the different parameters listed. N($\mu$,$\sigma$) and LN($\mu$,$\sigma$) stand for normal and lognormal distributions with mean $\mu$ and standard deviation $\sigma$, respectively. [Note that the implementations of most lognormal distributions require the parameters meanlog = $\log (\mu )-\log (1+\sigma ^2/\mu ^2)/2$ and sigmalog = $\sqrt{\log (1+\sigma ^2/\mu ^2}$)]

Variable	Prior distribution (units in Table 1)
K	LN (284.4, 57.6)
$Q_{\rm gw}$	LN (6, 1)
$\sigma _z$	LN (4.5, 0.45)
$\sigma _\xi$	LN (0.65, 0.3)
$S_0$	N (0, 5000) truncated to interval $[0, \infty )$
$\uplambda$	LN (0.1/60, 0.05/60)
$\xi _r$	N (0.5, 0.1)
$\gamma$	LN (0.5, 0.25)

### Dataset

To compare the different methods for stochastic model calibration, we use two different datasets from the same hydrological system, a small urban catchment located in Adliswil, Zurich, Switzerland. They differ in the accuracy of the rainfall measurements (Del Giudice et al. 2016). In the accurate scenario, termed “scenario 1” (Sc1), precipitation data results from the average rainfall measured at two point-scale pluviometers, both located in the immediate vicinity of the catchment. Time resolution is 1 min. Inaccurate “scenario 2” (Sc2) uses the rainfall recorded by a pluviometer operated by the Swiss meteorological office and located in Zurich Fluntern, which is about 6 km far apart from the catchment of interest. Data are stored every 10 min. Wastewater flow is measured at the outlet of the catchment with a time resolution of 4 min. These observations are the same for both scenarios. For this study, we choose to focus on a storm event that took place on June 10 2013 from about 18:00 to 20:00.

## Results and discussion

Since Scenario 2 is characterized by inaccurate rainfall measurements, it is numerically the more challenging one, and thus a stronger test of the different numerical approaches compared in this paper. Hence, we base our exposition primarily on this scenario. Nevertheless, we do the simulations also for scenario Sc1 for further validation.

### Scenario 2

#### Convergence of markov chains

To allow visual assessments of convergence for the tested inference methods, we plot the Markov chains for all the model parameters in Figs. 5, 6 and 7 in the Appendix. Overall, we conclude that the chains converge for all numerical approaches. For COUS, we run 4 independent Markov chains, each of length 200k steps, subsample them by a factor of 20 at run time to save storage space, and retain only the second half to pragmatically avoid initial state bias (Bacci et al. 2019). For HMC, we start from an initial 75k-step long chain (which we then discard as burn-in), and run 4 independent Markov chains each 100k-step long. For both COUS and HMC, the chains appear to be sufficiently long to ensure good mixing. For PMCMC, we use 48 chains, each collecting 3000 samples (with a burn-in of 1000 samples). Namely, to approximately obtain $10^5$ samples also for PMCMC, we use a much larger number of shorter chains. This is for three reasons. First, our PMCMC software implementation is parallel in nature. Second, differently from COUS, we use the EMCEE (Foreman-Mackey et al. 2013) sampler (and not the standard MCMC algorithm) to propagate the chains, which implies exchange of information between them, and hence a larger swarm has a positive effect on the speed of convergence. Third, the filtering step poses a sizeable overhead for models that run in the millisecond (or faster) timescales on a single CPU, as it is for our hydrological model. Hence, collecting many samples per chain is very time consuming, and this justifies resorting to a larger ensemble of shorter, communicating parallel chains.

To corroborate our intuition from Figs. 5, 6 and 7 regarding the amount of mixing in parameters space, we consider the average number of 25–$75\%$ crossings of the individual components of the Markov chains (Table 5). We define a 25–$75\%$ crossing as a transition from the lowest to the uppermost quartile of the sampled space (or the other way around). The average number of samples required to obtain such a crossing is comparable for the three methods (despite the thinning at run time that penalizes COUS in this analysis), and it is much shorter than the combined length of the chains.

In Fig. 8, we plot typical Markov chains for $\xi (t)$ evaluated at two different time-points, with and without rain observations. They appear to be well-mixed and show a good agreement between the three methods. However, the analogous plots for the rainfall in Fig. 9 exhibit some differences in the sampling of extreme values, in particular for PMCMC. For PMCMC, these differences might be explained by occasional filter collapses (see Figure S1 in the Supplementary Material) and by lack of smoothing in post processing.

As an additional convergence test for the state variables, we plot the 2.5-$-$97.5% envelopes of the stochastic dynamics and associated rainfall extracted from 4 independent and adjacent sampling blocks along a Markov chain (Fig. 10). COUS appears to be the method with the most consistent results. PMCMC shows the most rugged behavior, albeit quite consistently across the blocks. HMC shows a limited overlap only in the second half of the dynamics of the stochastic process, when rainfall is absent. However, since lack of overlap happens only at the lower end, the rainfall dynamics does not show any lack of homogeneity.

#### Marginal posteriors

We are interested in comparing the marginal posteriors of model parameters, model states, and discharge at the outlet of the catchment as obtained from the tested numerical approaches. We discuss these findings in the following paragraphs.

**Fig. 1:**
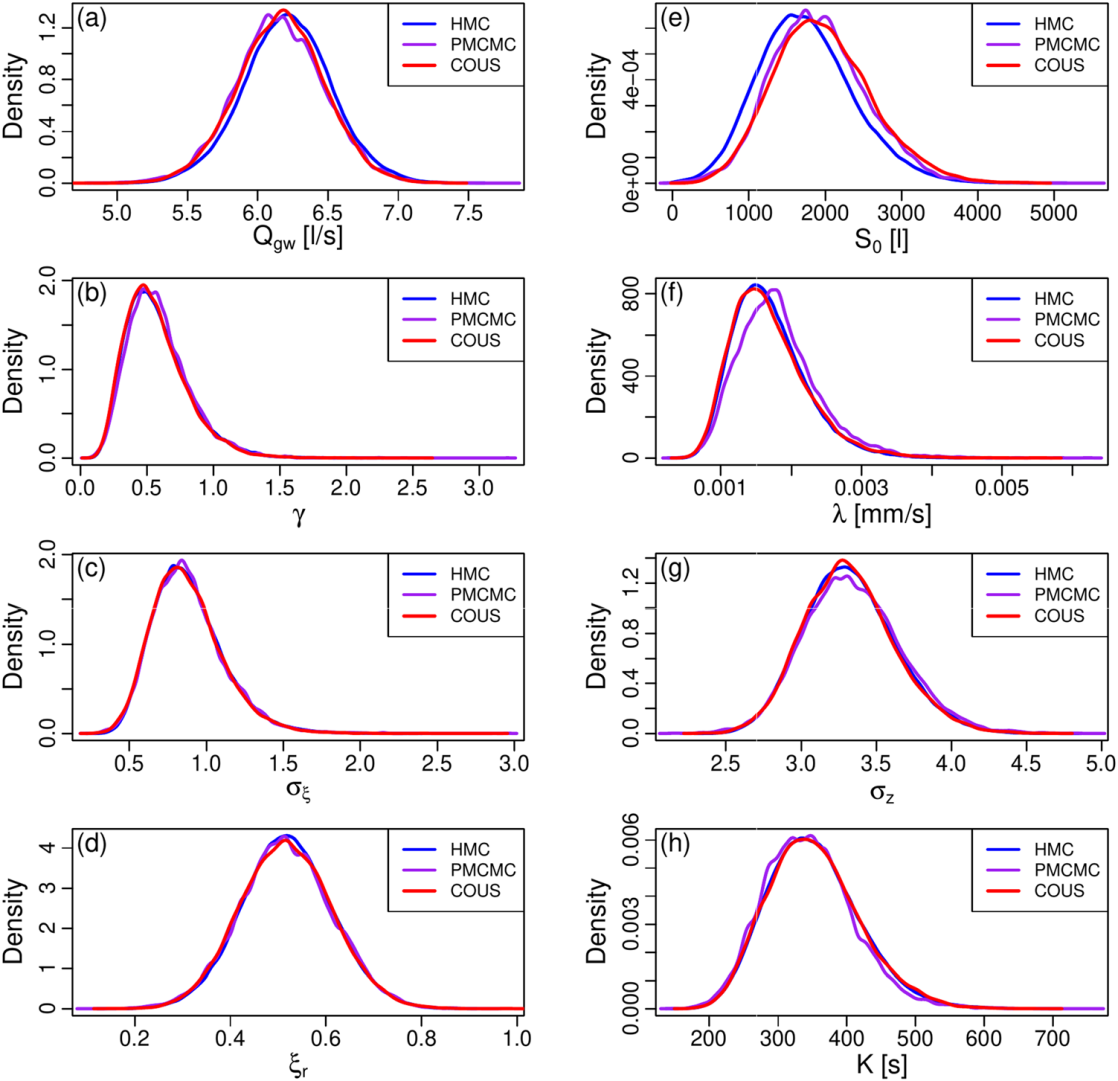
Parameters marginal posteriors, scenario 2. **a** Marginal posterior for $Q_{gw}$. **b** Marginal posterior for $\gamma$. **c** Marginal posterior for $\sigma _{\xi }$. **d** Marginal posterior for $\xi _r$. **e** Marginal posterior for $S_0$. **f** Marginal posterior for $\uplambda$. **g** Marginal posterior for $\sigma _z$. **h** Marginal posterior for *K*

Figure 1 shows the marginal posteriors of the model parameters for all three methods based on the respective sampling of the Markov chains, see Sect. 4.1.1. As it is apparent, the results are hardly distinguishable from one another, with empirical cumulative distributions differing almost everywhere by less than 4–5%, see Figs. S2–S3. These results also give us confidence in the numerical implementations of the three methods. The similarity between the marginal posteriors is indeed salient, even for parameters that are notoriously difficult to infer for this scenario, i.e., the ones related to the rainfall transformation $\gamma$, $\uplambda$, and $\xi _r$, and to the input observational error, $\sigma _\xi$. Noteworthy are also the 2D projections of the joint posterior distribution shown in Figs. S4–S6. Those look very similar across the methods, and also dissipate concerns regarding possible correlations between parameters (which might indicate an inadequate parameterization).

**Fig. 2:**
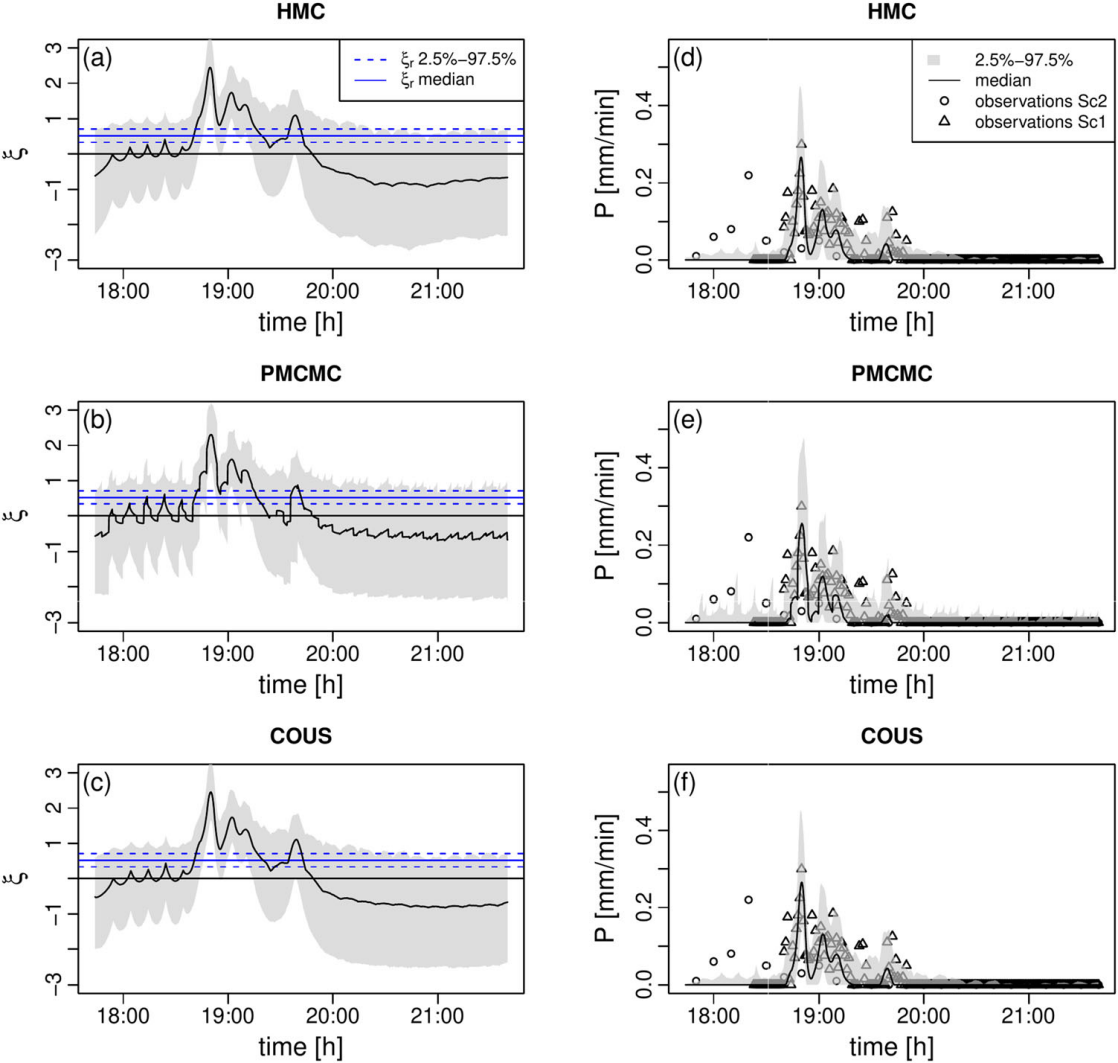
Stochastic process and rainfall posteriors, scenario 2. In all panels, the gray area defines the 2.5–97.5 percentile range. **a** Posterior of the stochastic process $\xi$ for HMC. **b** Same as (a) for PMCMC. **c** Same as (a) for COUS. **d** Posterior of the rainfall dynamics for HMC. **e** Same as (d) for PMCMC. **f** Same as (d) for COUS

For what concerns model states, as already discussed in Del Giudice et al. (2016), one key question is whether the use of the catchment outlet as an additional “rain gauge” can correct for input errors. Figure 2 shows the inferred marginal distributions of the time series of the stochastic process representing the rainfall potential as well as the corresponding rainfall intensity.

By visual inspection, the results look very similar across the methods. They also show that our inference methods are indeed able to correct for errors in the rain measurements. Due to the aforementioned convergence issues and lack of smoothing with PMCMC, the dynamics of the inferred stochastic process (Fig. 2b) appears more spiky than for HMC and COUS, albeit differences are not dramatic, and become even smaller when looking at the rainfall dynamics (Figs. 2d, e and f).

To further quantify the similarity in the stochastic process and rainfall dynamics across the methods, we calculate the density of these variables by binning on fixed bins at each time point when the selected dynamics has been computed and stored from the model simulations. These times are the same across all methods. We then take the difference of these densities across the methods, see Fig. S7 for the process $\xi$ and Fig. S8 for the rainfall. These differences are still at the resolution of the saved times and fixed bins, and are then normalized by the total density, which is invariant across time and method, due to the fixed number of counts and the fixed bins, respectively. This normalization also implies that the maximum theoretical difference (100%) would correspond to a scenario where all counts are concentrated in a bin for one method and that the compared method would have no counts in that bin. We note that the differences we obtain this way are generally very small (5–10%).

**Fig. 3:**
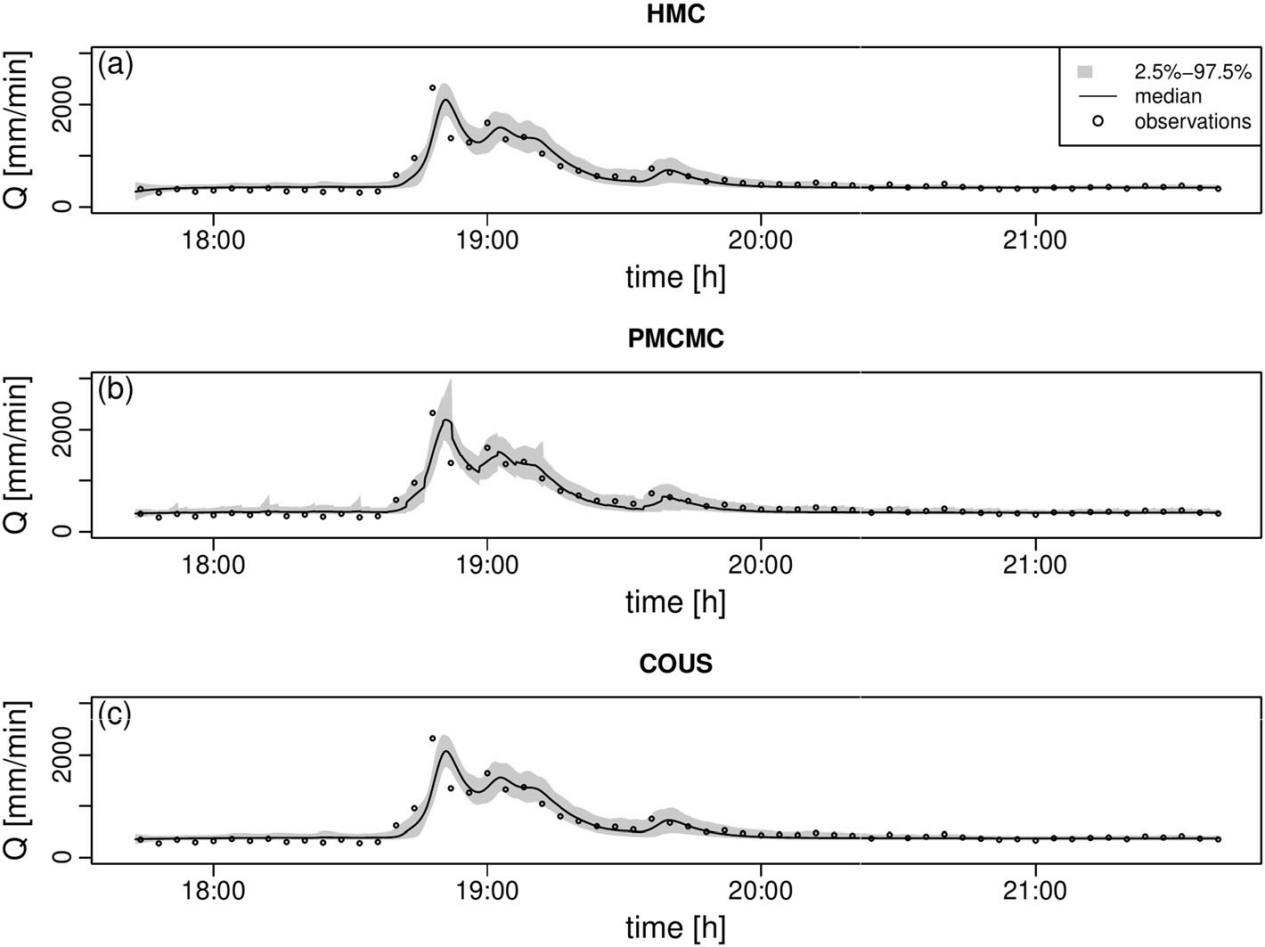
Posterior dynamics for the outflow *Q*, **scenario 2.** In all panels, the gray area defines the $2.5-97.5$ percentile range. **a** Posterior of the discharge *Q* for HMC. **b** Same as (a) for PMCMC. **c** Same as (a) for COUS

Finally, we look at the results for the discharge *Q* at the catchment outlet. We expect inference to be less difficult for discharge, as the observations are accurate. This statement is corroborated by the 2.5-$-$97.5% posterior intervals of the dynamics of *Q*, which look very similar across the methods, except for a few spikes for PMCMC, which could likely be leveled out by particles smoothing rather than filtering, see Fig. 3. Importantly, density analysis demonstrates that differences are small, Figure S9.

#### Efficiency

Table 3 shows the mean number of posterior evaluations (called number of function calls) per (multi- or uni-variate) effective sample. All methods attain a similar range of this metric, namely, between about 500 and 5500 function calls are required to obtain an independent parameter sample point. Two observations are worth discussing in more detail. First, while HMC appears to outperform the other two methods, the required differentiation of the energy function is considerably more expensive than just its evaluation, which is not reflected in Table 3. For our case study and implementation, this makes “function calls” for HMC about 5 times more time consuming than just the plain evaluation of the posterior. While this factor is not universal, it still would reduce the efficiency of HMC to some degree for any implementation. Second, PMCMC seems the method that offers the most homogeneous effective sample size values for the individual parameters.

**Table 3:** Mean number of function calls per effective sample, scenario 2. Either multi-variate estimation of the required mean number of posterior evaluations per effective sample (first row), or uni-variate estimation (per parameter) of the same metric

	HMC	PMCM	COUS
Multivariate	1315	2777	1818
*Uni-variate*			
$S_0$	671	5469	1697
*K*	925	5644	1827
$Q_{gw}$	1616	4841	1145
$\sigma _z$	1563	5608	1215
$\sigma _\xi$	1313	5194	1895
$\xi _r$	2245	5181	2341
$\gamma$	517	5710	1279
$\uplambda$	1505	5677	5650

### Scenario 1

**Fig. 4:**
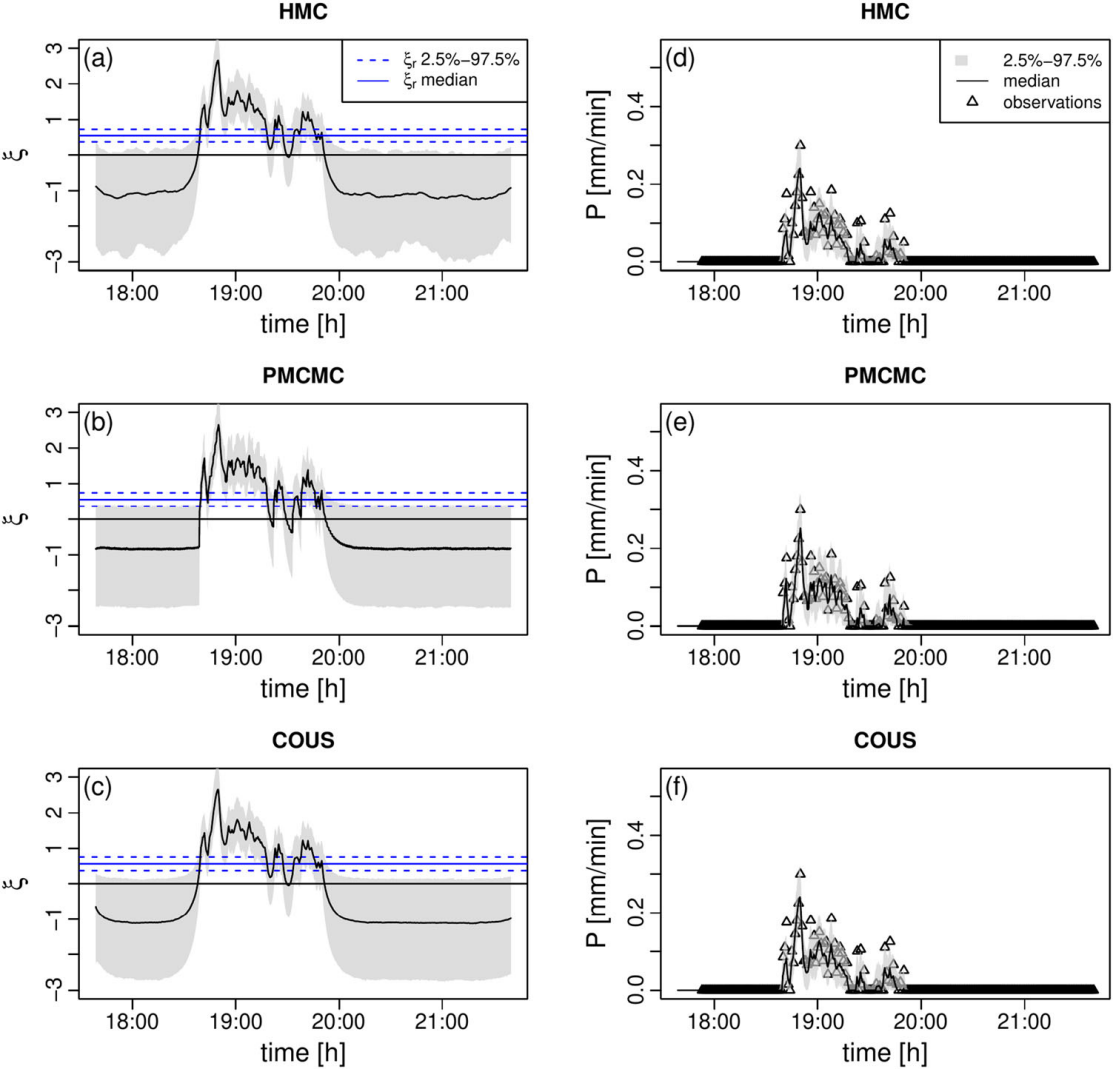
Stochastic process and rainfall posteriors, scenario 1. In all panels, the gray area defines the 2.5$-$97.5 percentile range. **a** Posterior of the stochastic process $\xi$ for HMC. **b** Same as (a) for PMCMC. **c** Same as (a) for COUS. **d** Posterior of the rainfall dynamics for HMC. **e** Same as (d) for PMCMC. **f** Same as (d) for COUS

**Fig. 5:**
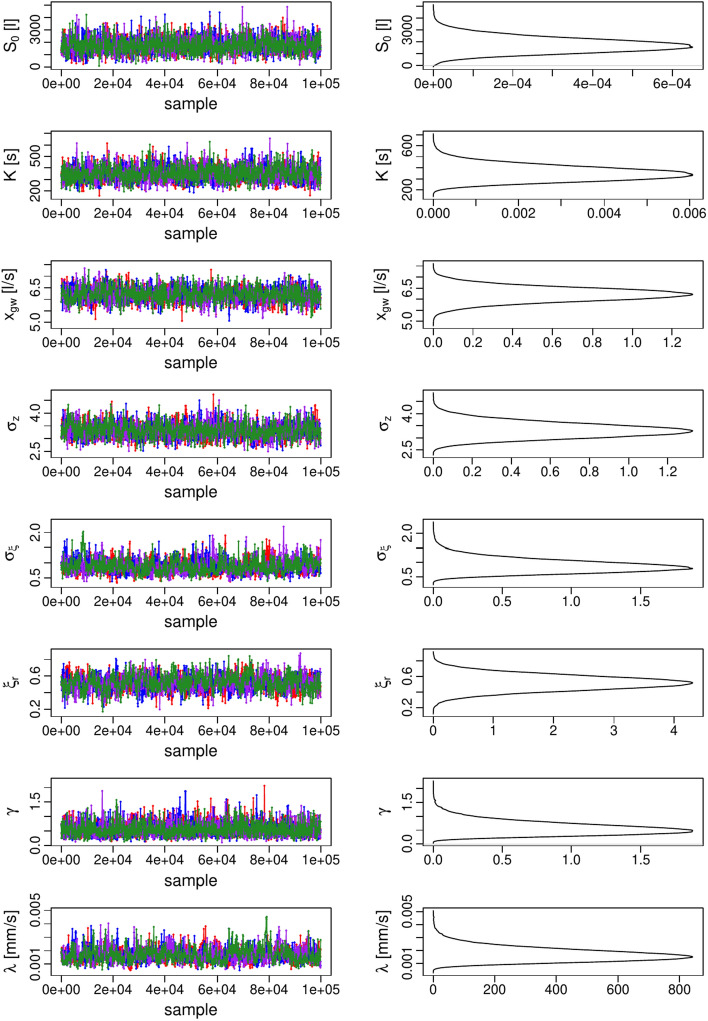
Markov chains and marginal posteriors for HMC, scenario 2.

**Fig. 6:**
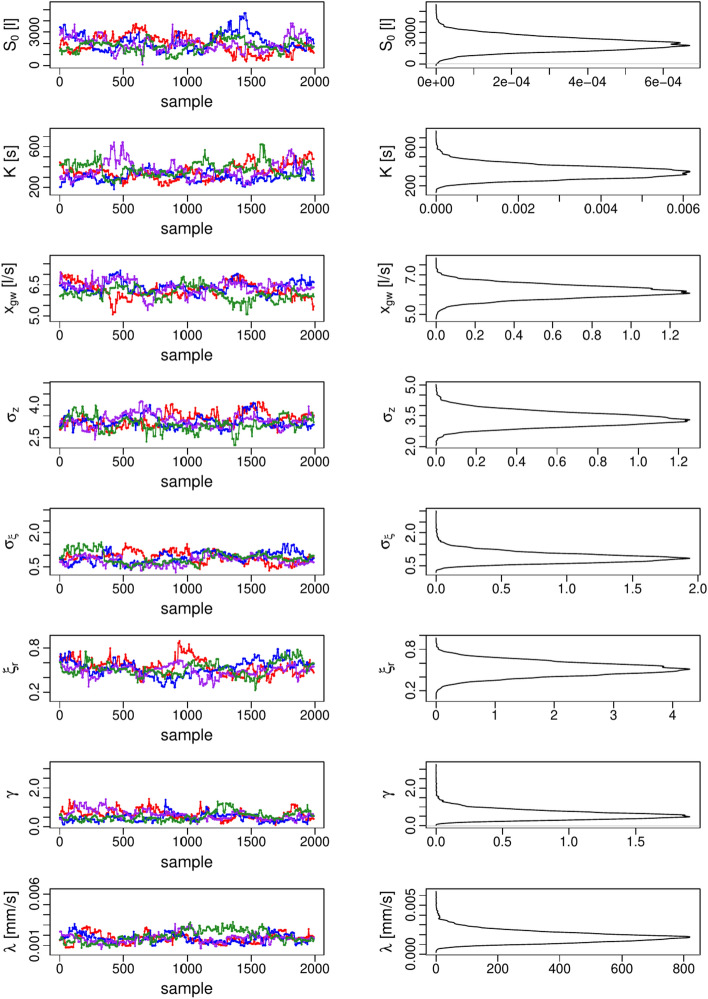
Markov chains and marginal posteriors for PMCMC, scenario 2.

**Fig. 7:**
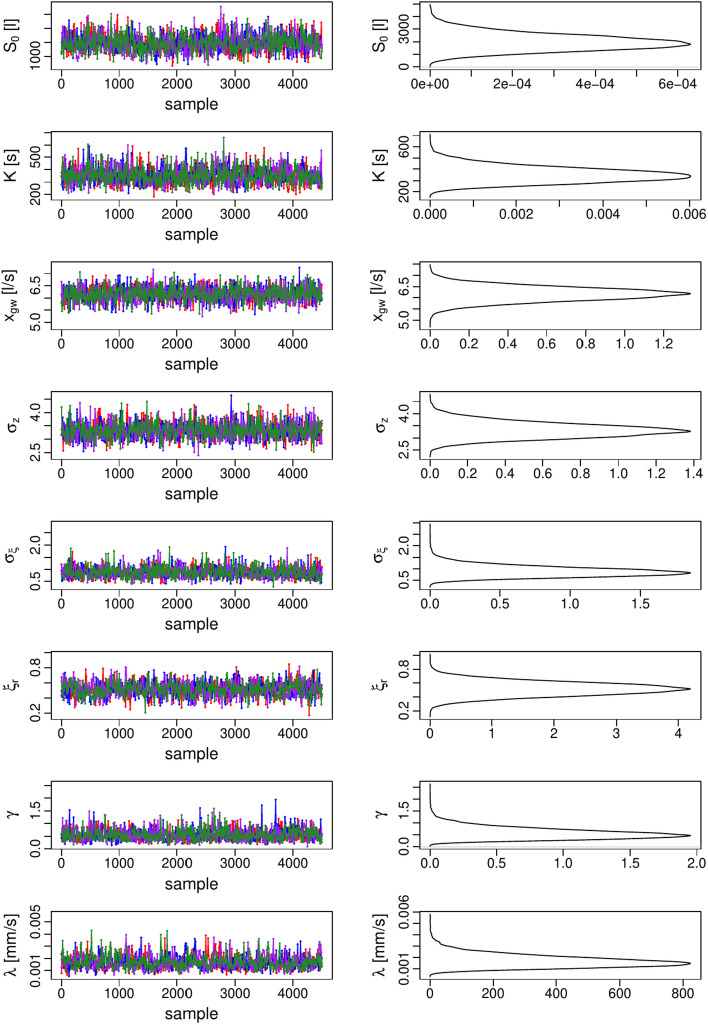
Markov chains and marginal posteriors for COUS, scenario 2 (subsampled by a factor of 20).

**Fig. 8:**
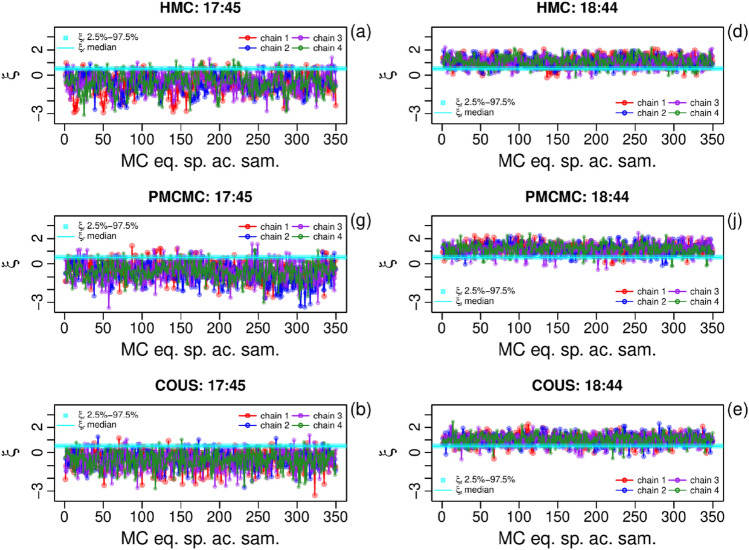
Stochastic process at fixed times for equally spaced accepted parameters samples, scenario 2. (a)–(f) are for times when no data is observed. (g)–(l) are for times when rainfall is observed

**Fig. 9:**
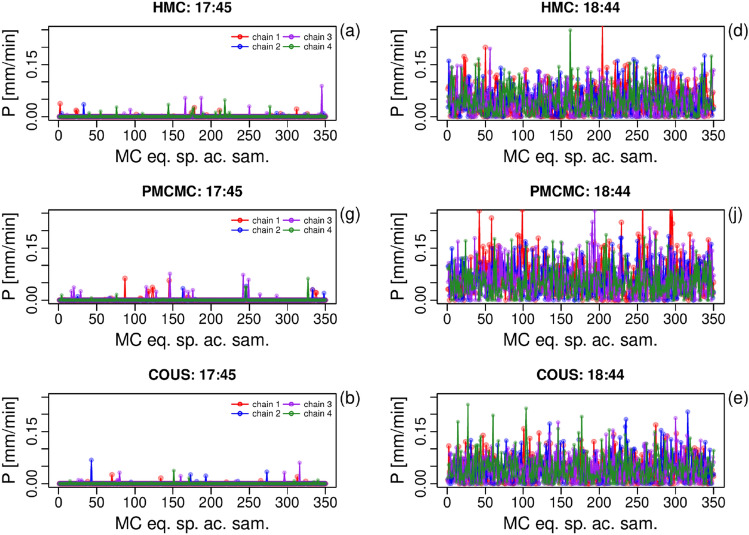
Rainfall at fixed times for equally spaced accepted parameters samples, scenario 2. (a)–(f) are for times when no data is observed. (g)–(l) are for times when rainfall is observed

**Fig. 10:**
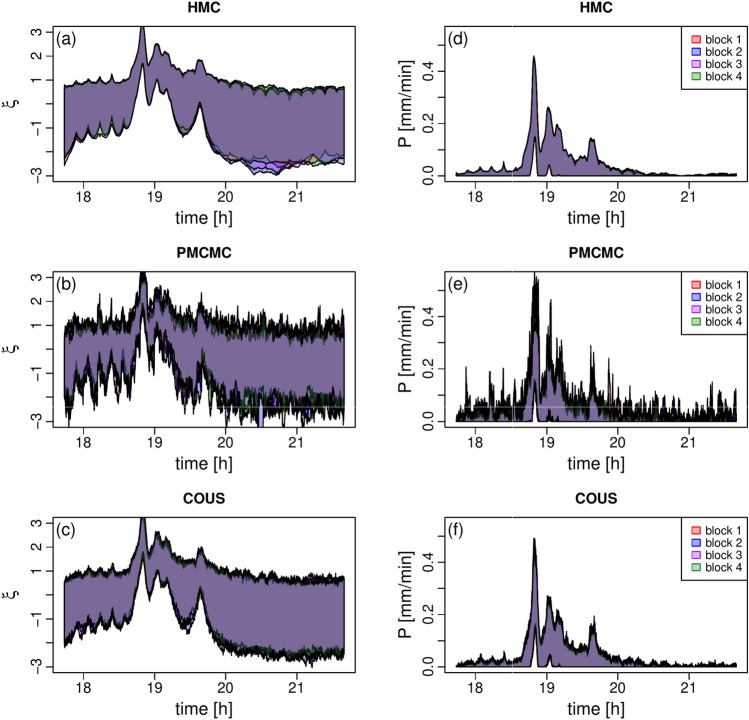
Process and rain overlaps for adjacent sampling blocks along one Markov chain, scenario 2. **(a)** Posterior blocks of the stochastic process $\xi$ for HMC. **(b)** Same as (a) for PMCMC. **(c)** Same as (a) for COUS. **(d)** Posterior blocks of the rainfall dynamics for HMC. **(e)** Same as (d) for PMCMC. **(f)** Same as (d) for COUS

Sc1 differs from Sc2 in that it offers more precise rainfall observations for inference (see Sect. 3.4). The main consequences are that the inferred stochastic process is smoother and the rainfall is obviously more in compliance with the observations used for the respective calibration, compare Fig. 4 with Fig. 2.

As expected for all methods, the dynamics of the stochastic process and rainfall seem robust and converged, see Figure S10, and the larger availability of data has a positive effect on filter collapses, compare Figure S11 with Figure S1.

The inferred dynamics of the discharge shows small uncertainty as well, with some rugged behavior from PMCMC and stronger agreement between HMC and COUS, Figure S12.

The Markov chains do not raise any concern regarding convergence, see Figs. S13–S15, and the marginal posteriors of the parameters are in good agreement across the approaches, see Figs. S16–S18. However, it is noticeable that the initial volume of water in the reservoir, $S_0$, is more uncertain for PMCMC in Sc1 than in Sc2. It is unclear why this is the case, although it is likely that the more precise estimation of the rainfall makes the model less sensitive to this parameter. However, we should also note that 2D projections of the parameters joint posterior show a weaker correlation between $S_0$ and *K* for PMCMC, see Figs. S19–S21, the consequence of which is in fact the larger spread of $S_0$ values.

Performance metrics related to sampling, such as the number of Markov chains inter-quartile trips, Table 6, and the number of function calls per effective sample, Table 7, are in agreement with what already shown for Sc2. Namely, PMCMC with the EMCEE sampler appears to be the method with the most consistent statistics, with performance being comparable across all methods.

## Summary and conclusions

Table 4 compares conceptual aspects and numerical efficiency of the three algorithms. Together with the numerical results discussed in the previous section, we come to the following conclusions.

**Table 4:** Summary comparison of the three numerical techniques

Criterion	HMC	PMCMC	COUS
Extent of modifications to user’s simulation program	Implementation with automatic differentiation needed	Step by step integration from output to output and re-initialization of particles	Integration with prescribed time series for inputs or parameters needed
Universality	Any stochastic process with continuous variables	Any stochastic process, also with discrete states	Only Ornstein-Uhlenbeck process (and transformations)
Key tuning needs	Masses, time-step	Number of particles	Number of sub-intervals
Efficiency	Highest	Lowest	Intermediate

- All three tested algorithms can successfully be applied to our inference problem of inferring the stochastic rainfall process jointly with constant model parameters. They lead, up to small numerical inaccuracies, to the same results, consistently and robustly. Besides this proof of concept, this is also a very strong indication for correct implementations, which is an often overlooked challenge in complex inference problems. Multiple, independent implementations are extremely helpful to identify implementation problems.
- There is a clear trade-off between implementation effort (smallest for COUS, largest for HMC) and universality (largest for PMCMC and HMC, smallest for COUS), see Table 4.
- The numerical efficiency of the alternative approaches is much more difficult to compare, as it depends strongly on the inference problem and relevant implementation, on the tuning parameters of the inference algorithms, and on the possible parallelization scheme of the dedicated software, which is most natural for PMCMC, as it requires the use of an ensemble of communicating particles by design. For our case study, there is not a dramatic difference in effective sample size per evaluation of the posterior, with a small advantage for HMC over COUS, and with PMCM trailing the other two methods by a factor of 1.5–2.5. However, especially for HMC, the comparison in Table 3 does not fully reflect computational time due to the fact that the computation of the gradients is likely to always be more expensive than the evaluation of the energy. Yet, these results indicate another trade-off between implementation effort and efficiency.

Overall, we recommend a step-by-step process to select the inference algorithm.

1. First, the applicability of the algorithms has to be checked (see Table 4). Among the investigated algorithms, we have to go for PMCMC if the states of the stochastic processes are discrete (e.g., numbers of organisms in ecological models). If we need a high flexibility in the stochastic processes, we can choose among HMC and PMCMC. Finally, if it suffices to use poentially transformed Ornstein-Uhlenbeck processes, we can choose among all three algorithms.
2. If we still have remaining choices, we can consider implementation aspects. In most cases, coupling the model to COUS will be fastest to implement, as this only requires the simulation program to accept a prescribed (by the inference algorithm) time series of model parameters or input (which will be realizations of the Ornstein-Uhlenbeck stochastic processes). PMCMC requires piece-by-piece evaluation of the model time series, which should also not require a very high implementation effort. However, the need to resample the particles according to the likelihood of the observations might not be straightforward, and can easily become the bottleneck of numerical performances with fast models. Finally, HMC will require a re-implementation of the model on a platform that allows for automatic differentiation, unless the model is already implemented on such a platform, or will require the implementation of the differentiation of the energy function and relevant time-integration, which might be difficult.
3. Third, if there are still remaining choices, efficiency can be considered, which in most cases will favor HMC.

### Supplementary Information

Below is the link to the electronic supplementary material.

Supplementary file 1 (pdf 6796 KB)

## Data Availability

The PMCMC algorithm has been implemented in the SPUX framework, the documentation of which is found at https://spux.readthedocs.io/en/latest/. The COUS algorithm can be found in the R package timedeppar, published at https://cran.r-project.org/package=timedeppar. The C++ code for HMC is available on Github at https://github.com/ulzegasi/HMC_SIP.git. The specific versions and input files used for this study and relevant inputs are available at https://drive.switch.ch/index.php/s/sbGyuJy62TAH5yU.

## Appendix

See Figs. 5, 6, 7, 8, 9 and 10 and See Tables 5, 6 and 7

**Table 5:** Mean number of posterior samples to achieve an inter-quartile crossings per method and parameter for the MC sampling, scenario 2. COUS values are likely overestimated due to thinning at run time

Parameter	HMC	PMCMC	COUS
$Q_{gw}$	227	275	207
$S_0$	101	259	208
$\gamma$	70	254	216
$\uplambda$	172	244	576
$\sigma _\xi$	147	255	248
$\sigma _z$	215	267	196
$\xi _r$	313	265	390
*K*	126	266	264

**Table 6:** Mean number of posterior samples to achieve an inter-quartile crossings per method and parameter for the MC sampling, scenario 1. COUS values are likely overestimated due to thinning at run time

Parameter	HMC	PMCMC	COUS
$Q_{gw}$	176	274	146
$S_0$	69	282	141
$\gamma$	15	274	167
$\uplambda$	104	276	502
$\sigma _\xi$	39	271	313
$\sigma _z$	212	273	162
$\xi _r$	45	283	421
*K*	58	300	162

**Table 7:** Mean number of function calls per effective sample, scenario 1. Either multi-variate estimation of the required mean number of posterior evaluations per effective sample (first row), or uni-variate estimation (per parameter) of the same metric

	HMC	PMCM	COUS
Multivariate	1298	2564	1420
*Uni-variate*			
$S_0$	477	5360	841
*K*	638	5434	967
$Q_{gw}$	1177	5249	817
$\sigma _z$	1397	5157	939
$\sigma _\xi$	3489	4975	2884
$\xi _r$	3708	5118	4566
$\gamma$	266	4986	965
$\uplambda$	5439	5392	5149

## Footnotes

1 For simplicity, we assume here a single scalar state variable $\xi (t)$.
