# Supplementary material for: A comparison of numerical approaches for statistical inference with stochastic models
Source: Stoch Environ Res Risk Assess. 2023 Apr 13;37(8):3041–61. doi: 10.1007/s00477-023-02434-z (PMC10368571; doi:10.1007/s00477-023-02434-z)
Supplement: Supplementary file 1 — (pdf 6796 KB) [file 477_2023_2434_MOESM1_ESM.pdf]

# Supporting Information to A Comparison of Numerical Approaches to Statistical Inference in Stochastic Models

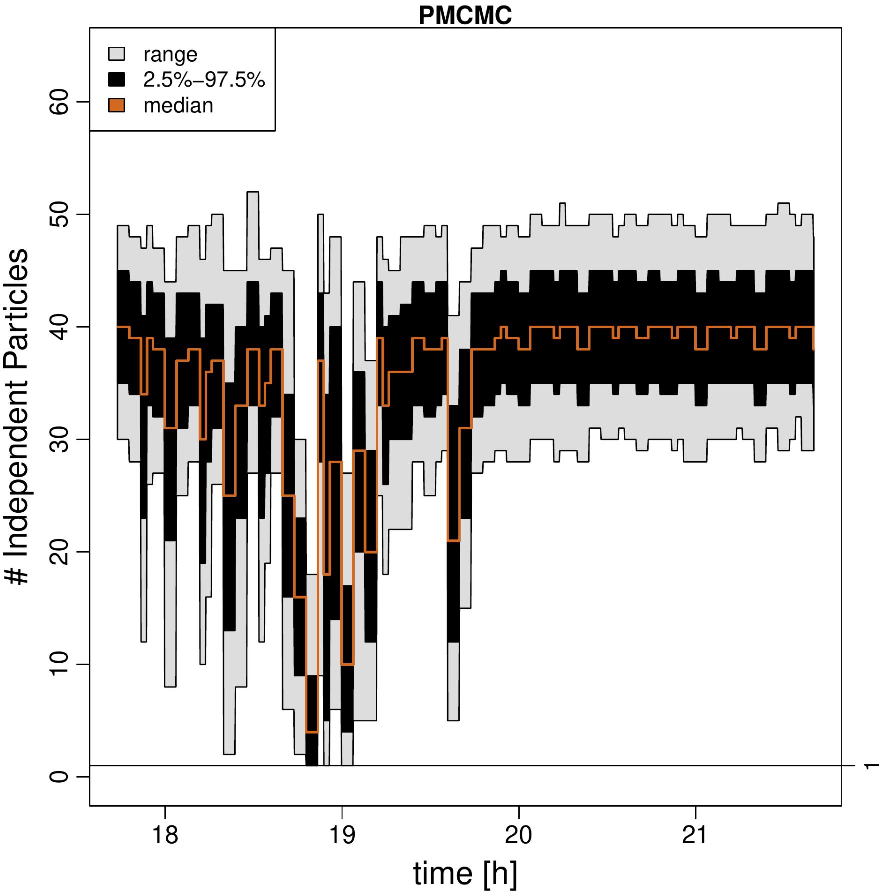


**Fig. S1** **Number of independent particles along the time series, PMCMC, scenario 2.**

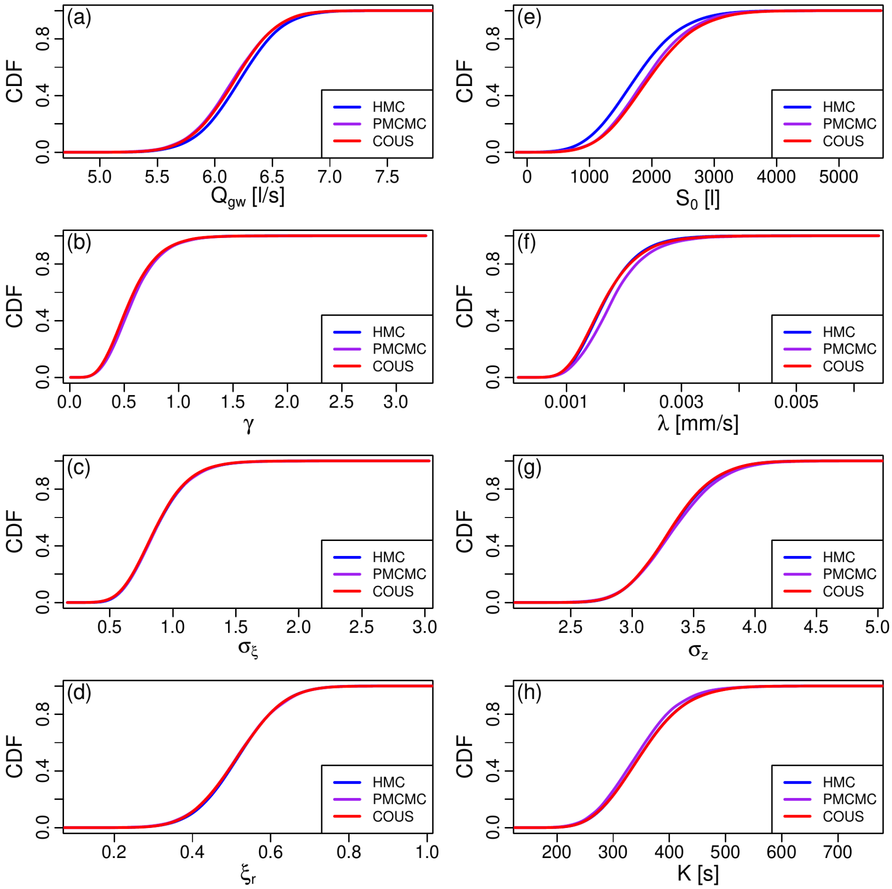


**Fig. S2 Empirical cumulative distributions of parameters marginal posteriors, Sc2. (a)** Marginal posterior cumulative distribution for $Q_{gw}$. **(b)** Marginal posterior cumulative distribution for $\gamma$. **(c)** Marginal posterior cumulative distribution for $\sigma_{\xi}$. **(d)** Marginal posterior cumulative distribution for $\xi_r$. **(e)** Marginal posterior cumulative distribution for $S_0$. **(f)** Marginal posterior cumulative distribution for $\lambda$. **(g)** Marginal posterior cumulative distribution for $\sigma_z$. **(h)** Marginal posterior cumulative distribution for $K$.

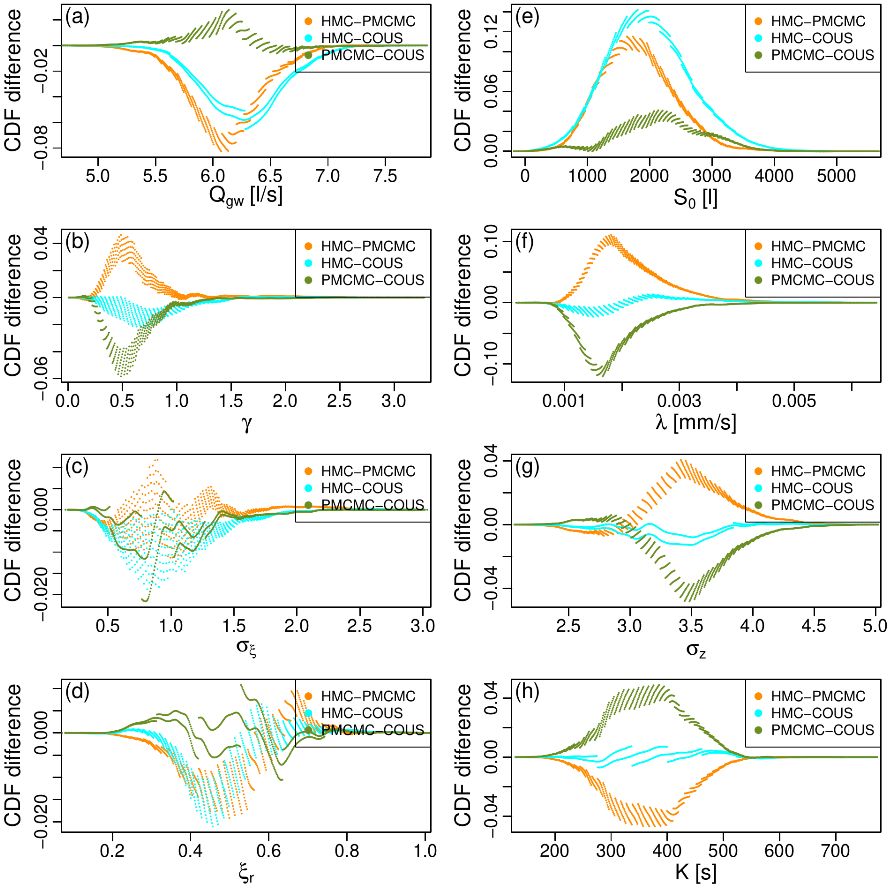


**Fig. S3 Differences in the empirical cumulative distributions of parameters marginal posteriors, Sc2. (a)** Differences in the marginal posterior cumulative distribution for $Q_{gw}$. **(b)** Differences in the marginal posterior cumulative distribution for $\gamma$. **(c)** Differences in the marginal posterior cumulative distribution for $\sigma_\xi$. **(d)** Differences in the marginal posterior cumulative distribution for $\xi_r$. **(e)** Differences in the marginal posterior cumulative distribution for $S_0$. **(f)** Differences in the marginal posterior cumulative distribution for $\lambda$. **(g)** Differences in the marginal posterior cumulative distribution for $\sigma_z$. **(h)** Differences in the marginal posterior cumulative distribution for $K$.

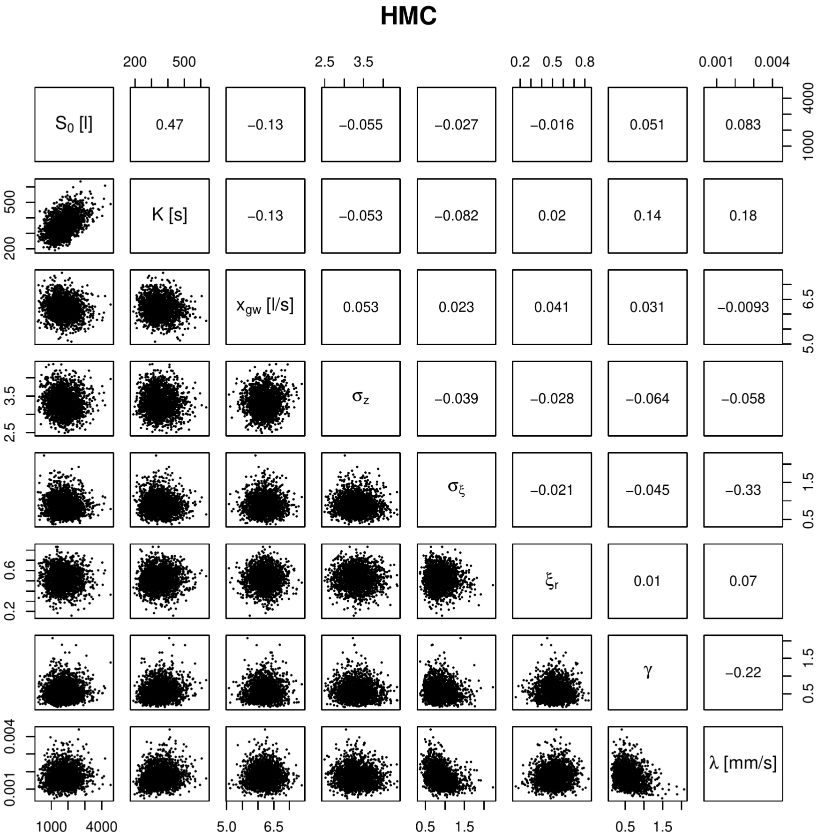


**Fig. S4** **2D marginal posteriors of parameters for HMC, scenario 2.**

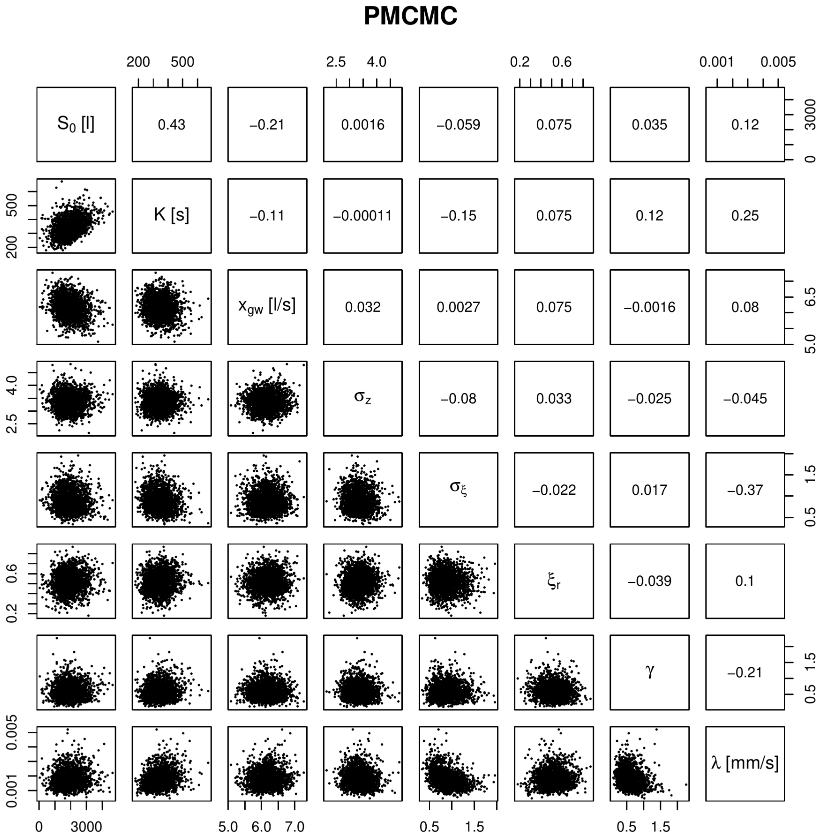


**Fig. S5** **2D marginal posteriors of parameters for PMCMC, scenario 2.**

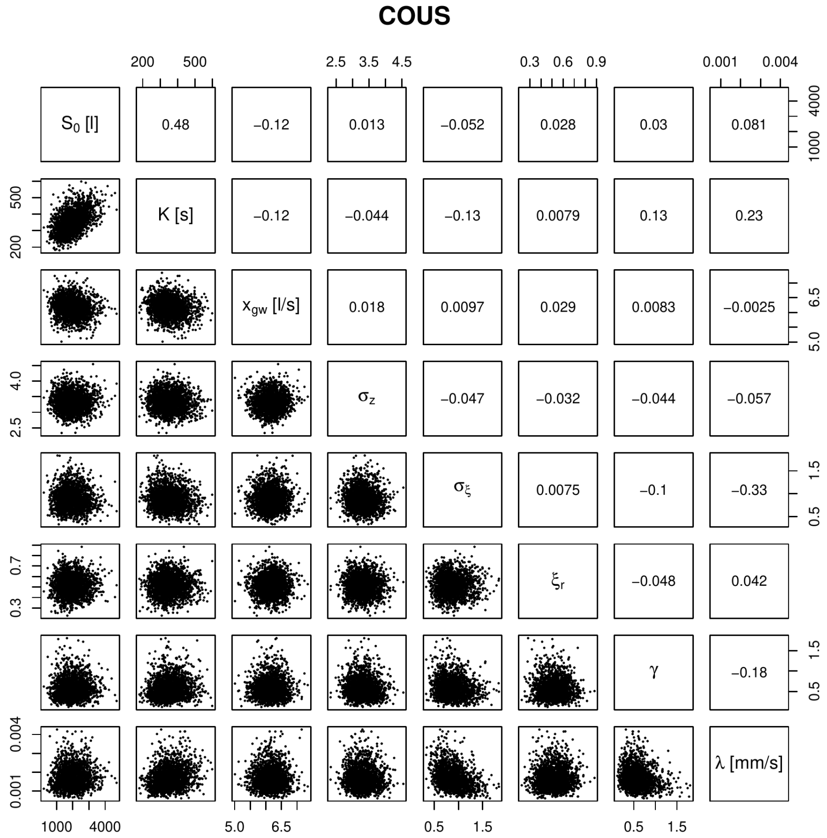


**Fig. S6** **2D marginal posteriors of parameters for COUS, scenario 2.**

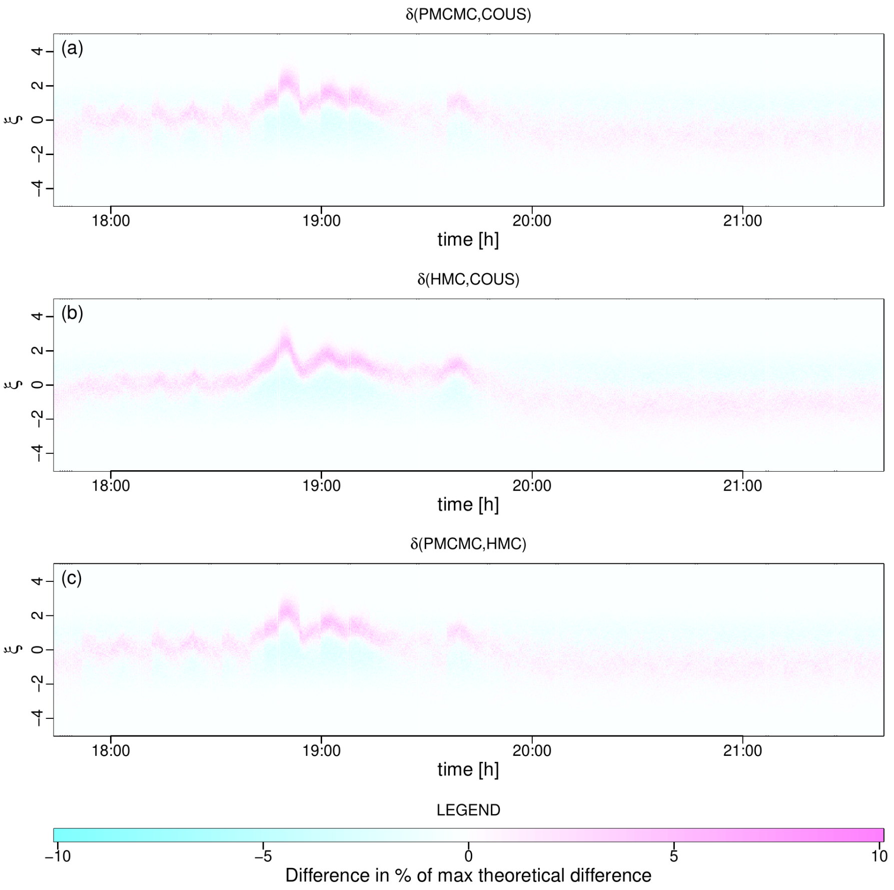


**Fig. S7 Normalized difference between the stochastic process $\xi$ density at each available computing time, scenario 2.** (a) Difference between PMCM and COUS results. (b) Difference between HCM and COUS results. (c) Difference between PMCM and HMC results.

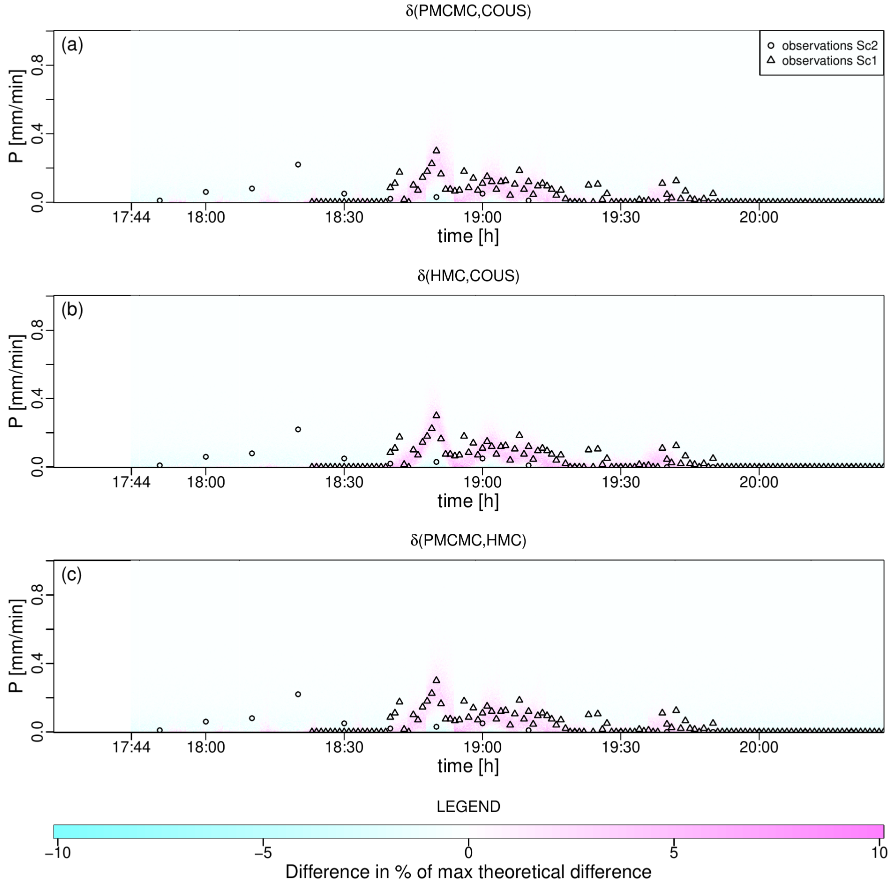


**Fig. S8 Normalized difference between the rainfall density at each available computing time, scenario 2.** (a) Difference between PMCM and COUS results. (b) Difference between HCM and COUS results. (c) Difference between PMCM and HMC results.

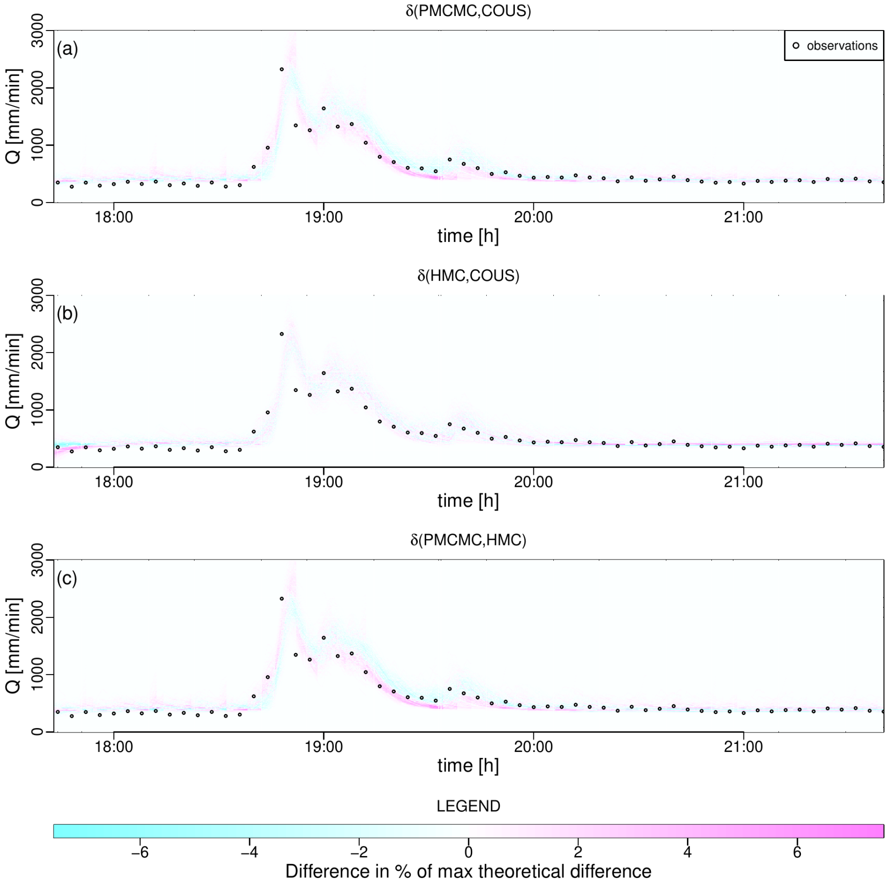


**Fig. S9 Normalized difference between the discharge density at each available computing time, scenario 2.** (a) Difference between PMCM and COUS results. (b) Difference between HCM and COUS results. (c) Difference between PMCM and HMC results.

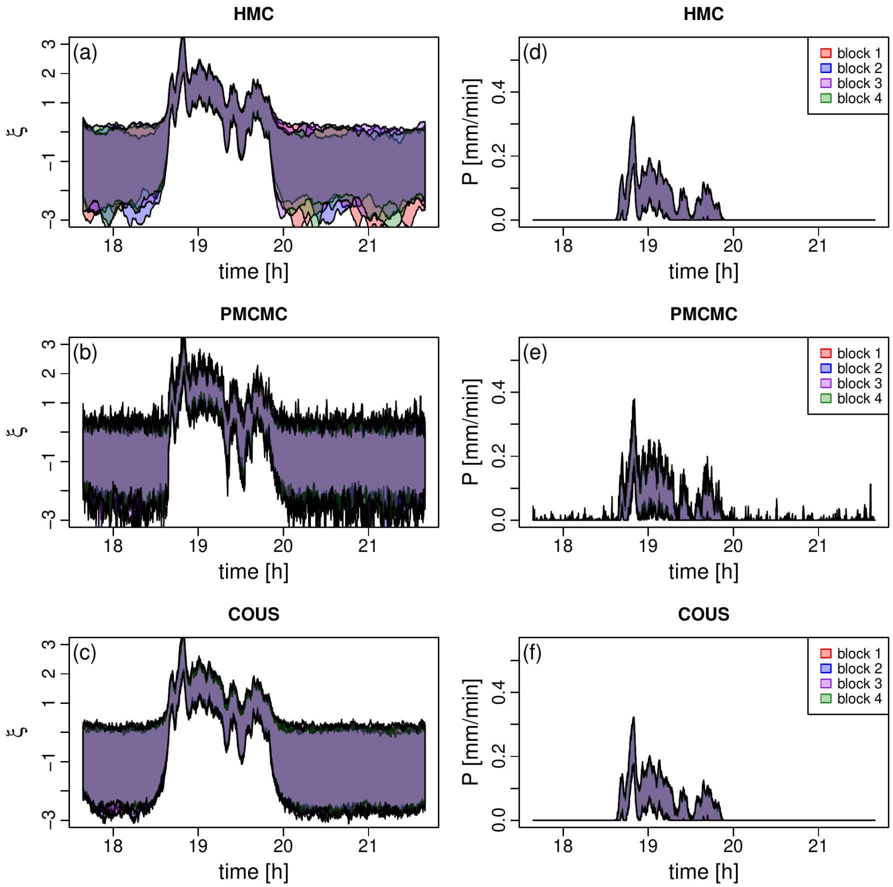


**Fig. S10 Process and rain overlaps for adjacent sampling blocks along one Markov chain, scenario 1.**

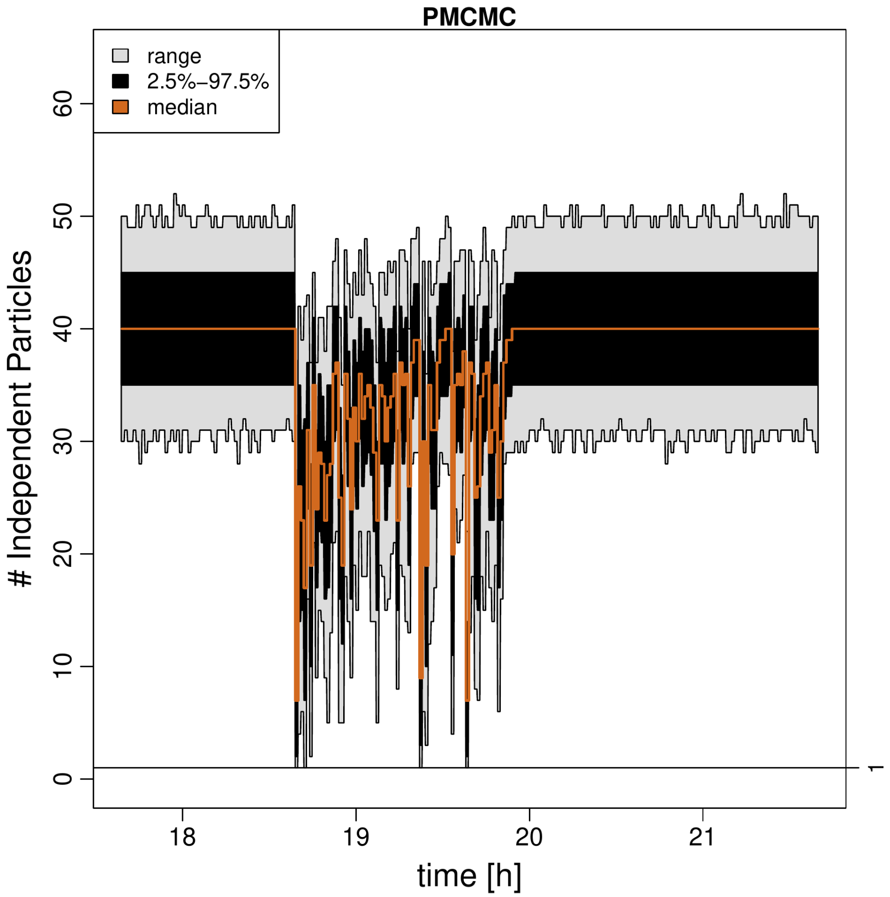


**Fig. S11** **Number of independent particles along the time series, PMCMC, scenario 1.**

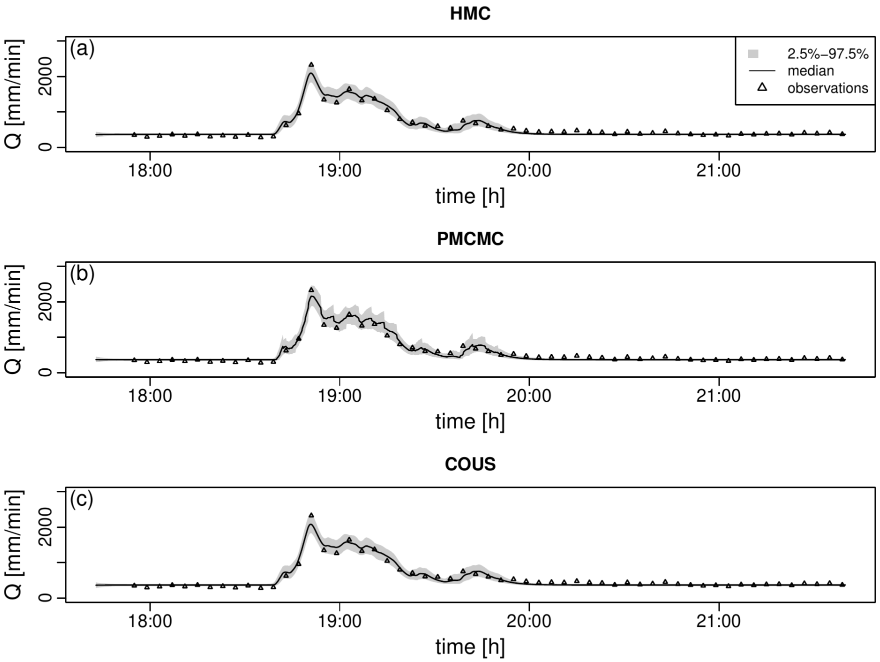


**Fig. S12 Discharge posterior, scenario 1. (a)** Posterior of the discharge $Q$ for HMC. **(b)** Same as (a) for PMCMC. **(c)** Same as (a) for COUS.

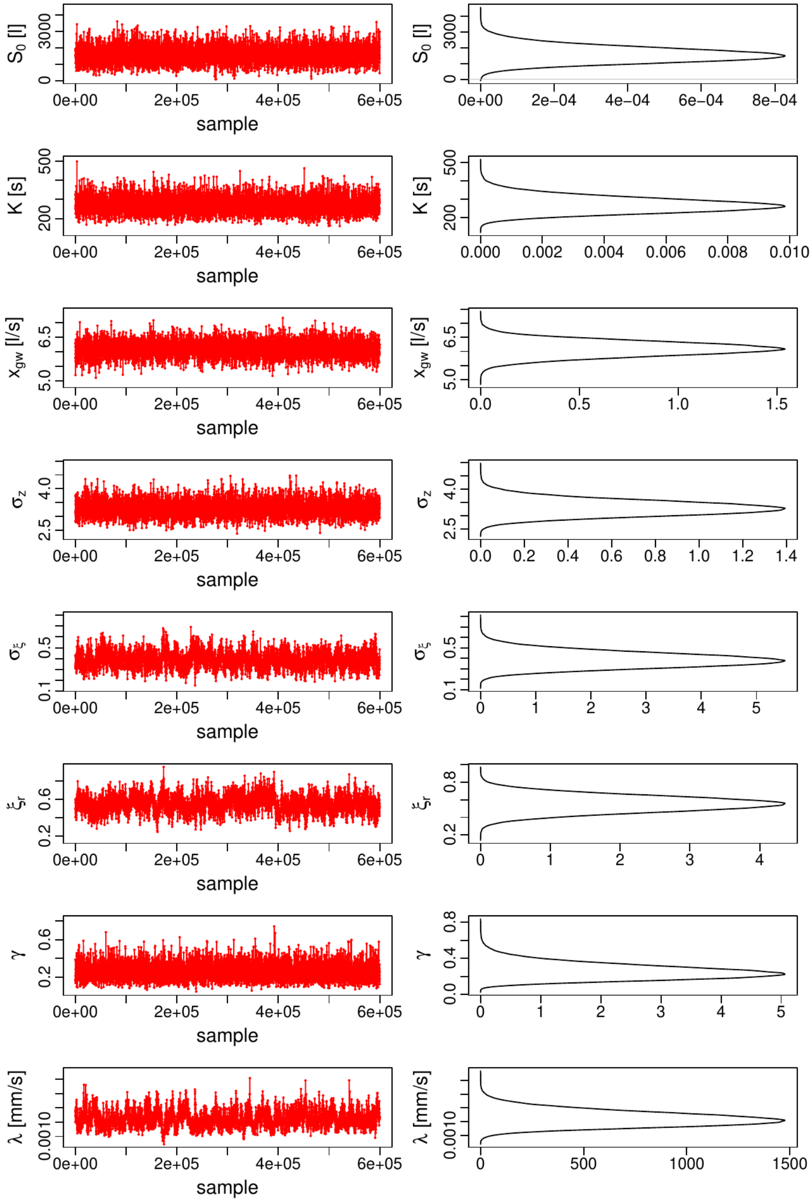


**Fig. S13 Markov chains and marginal posteriors for HMC, scenario 1.**

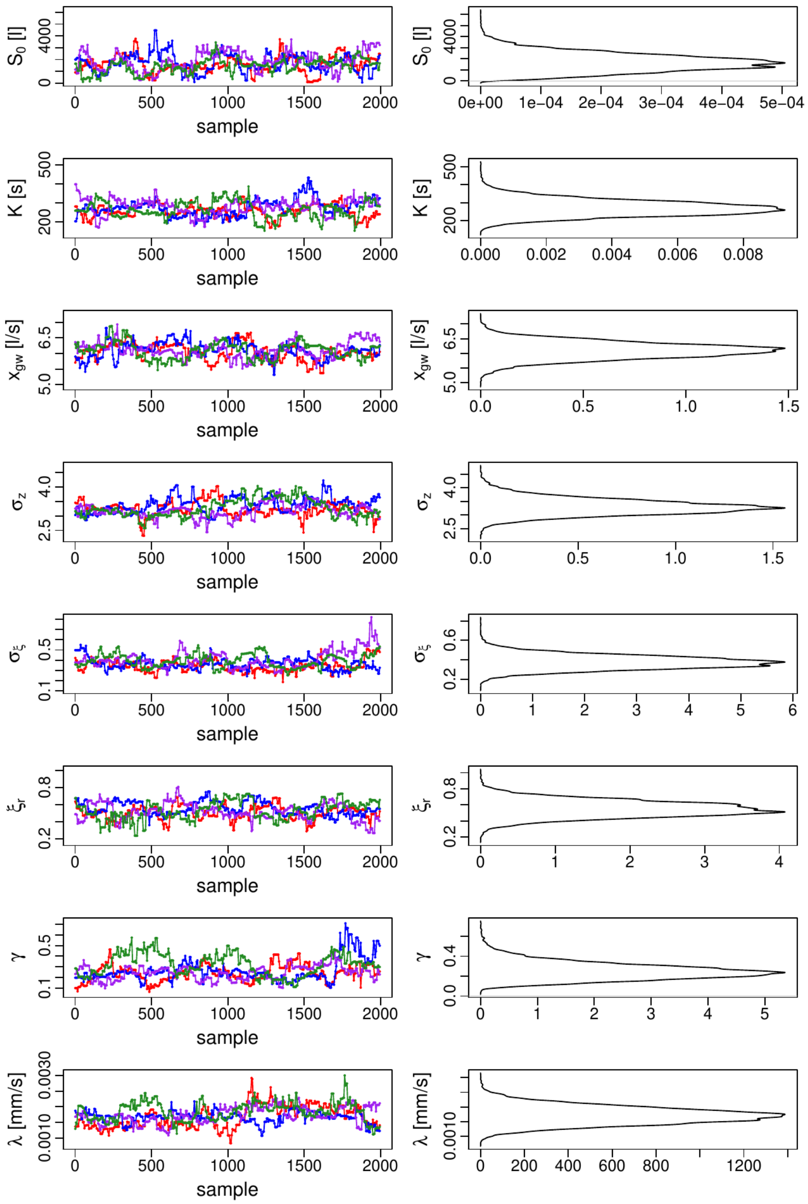


**Fig. S14** **Markov chains and marginal posteriors for PMCMC, scenario 1.**

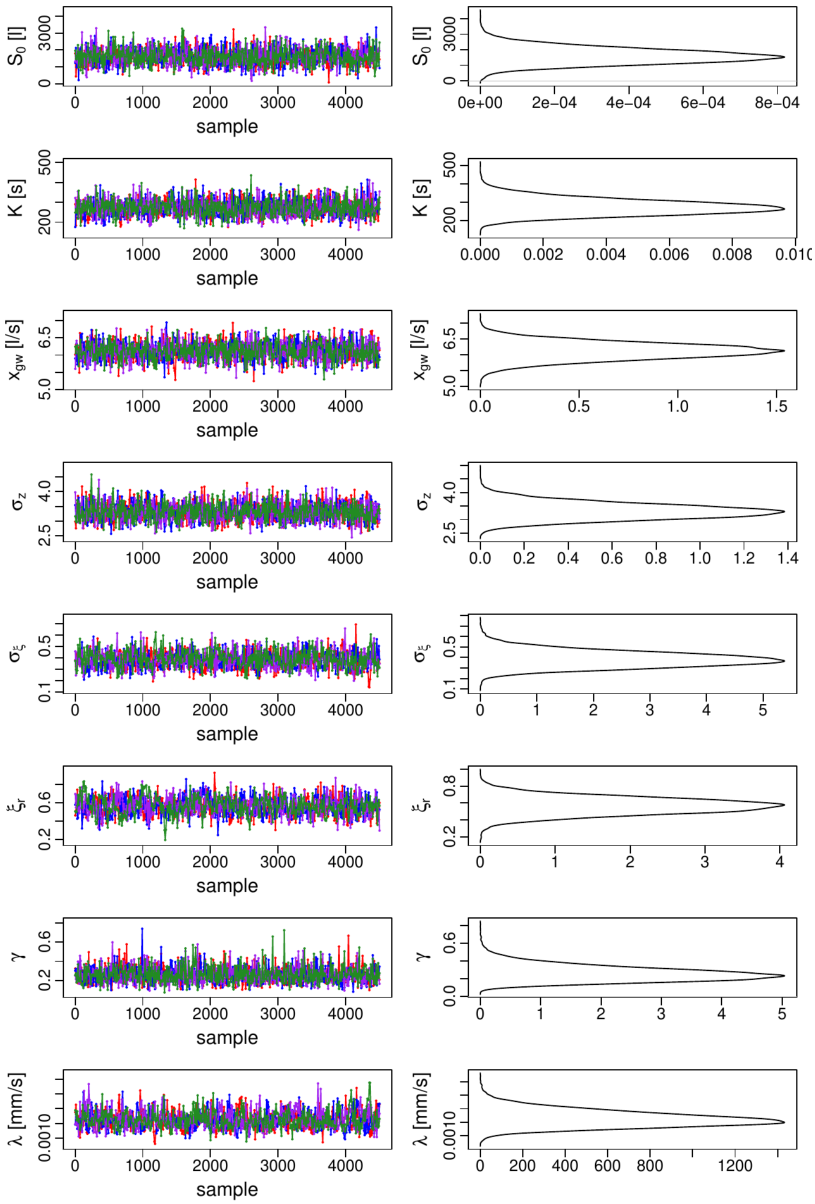


**Fig. S15 Markov chains and marginal posteriors for COUS, scenario 1.**

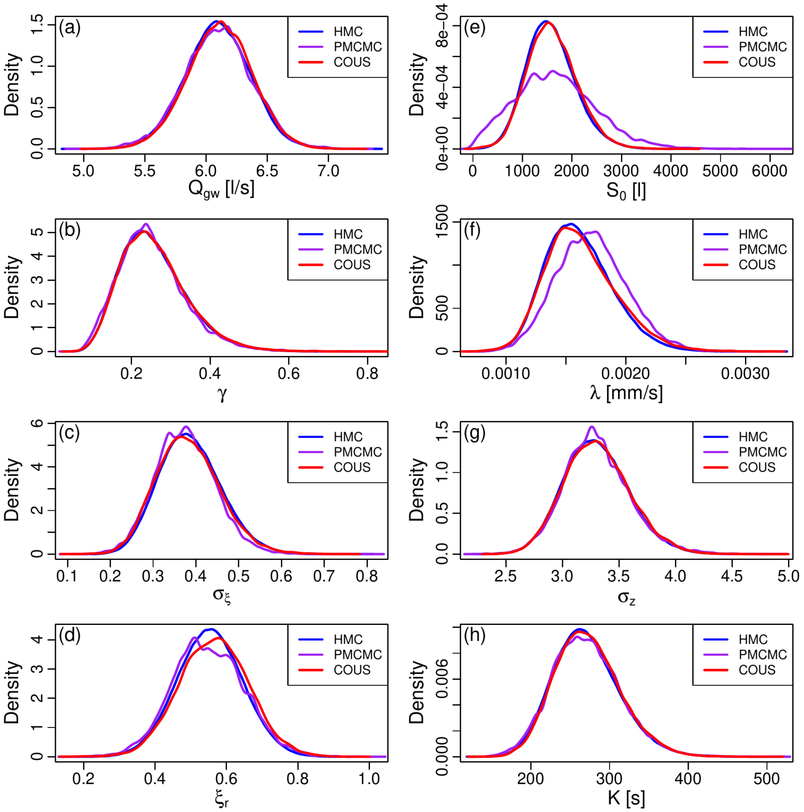


**Fig. S16 Parameters marginal posteriors, scenario 1. (a)** Marginal posterior for $Q_{gw}$. **(b)** Marginal posterior for $\gamma$. **(c)** Marginal posterior for $\sigma_\xi$. **(d)** Marginal posterior for $\xi_r$. **(e)** Marginal posterior for $S_0$. **(f)** Marginal posterior for $\lambda$. **(g)** Marginal posterior for $\sigma_z$. **(h)** Marginal posterior for $K$.

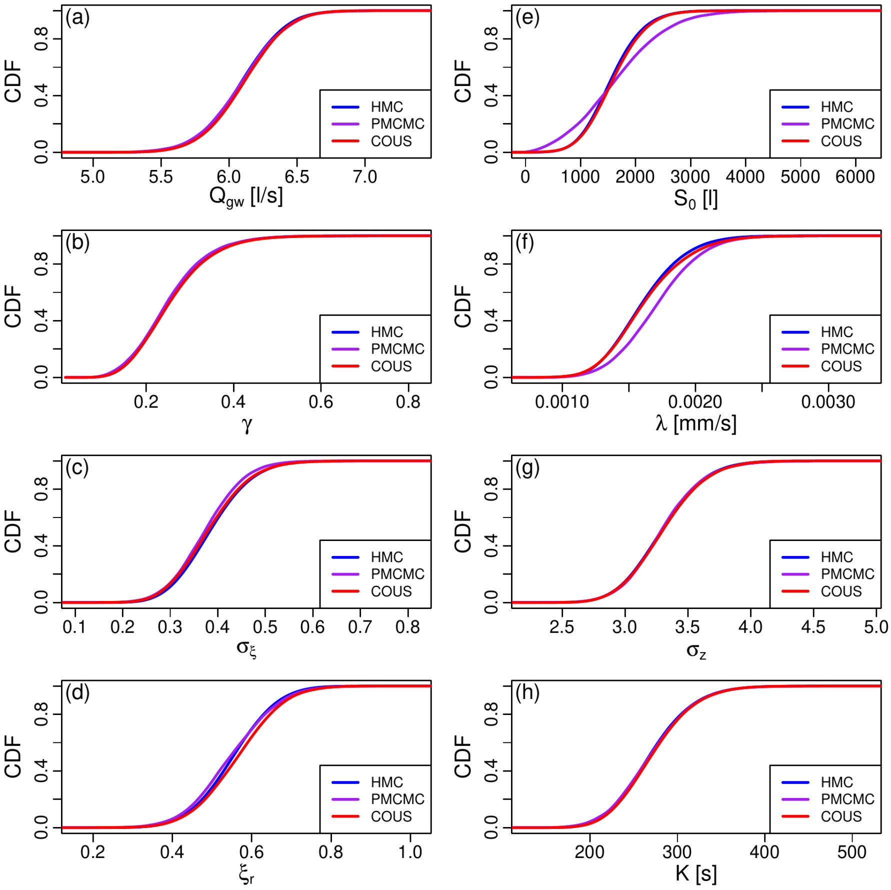


**Fig. S17 Empirical cumulative distributions of parameters marginal posteriors, Sc1.** **(a)** Marginal posterior cumulative distribution for $Q_{gw}$. **(b)** Marginal posterior cumulative distribution for $\gamma$. **(c)** Marginal posterior cumulative distribution for $\sigma_{\xi}$. **(d)** Marginal posterior cumulative distribution for $\xi_r$. **(e)** Marginal posterior cumulative distribution for $S_0$. **(f)** Marginal posterior cumulative distribution for $\lambda$. **(g)** Marginal posterior cumulative distribution for $\sigma_z$. **(h)** Marginal posterior cumulative distribution for $K$.

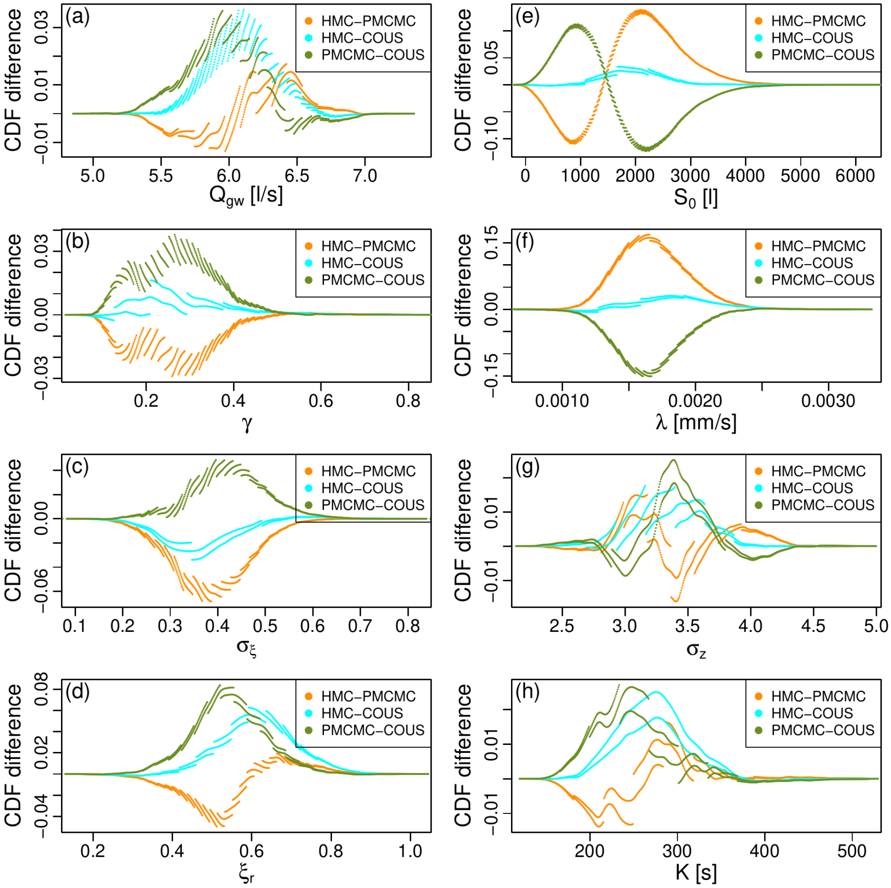


**Fig. S18 Differences in the empirical cumulative distributions of parameters marginal posteriors, Sc1. (a)** Differences in the marginal posterior cumulative distribution for $Q_{gw}$. **(b)** Differences in the marginal posterior cumulative distribution for $\gamma$. **(c)** Differences in the marginal posterior cumulative distribution for $\sigma_\xi$. **(d)** Differences in the marginal posterior cumulative distribution for $\xi_r$. **(e)** Differences in the marginal posterior cumulative distribution for $S_0$. **(f)** Differences in the marginal posterior cumulative distribution for $\lambda$. **(g)** Differences in the marginal posterior cumulative distribution for $\sigma_z$. **(h)** Differences in the marginal posterior cumulative distribution for $K$.

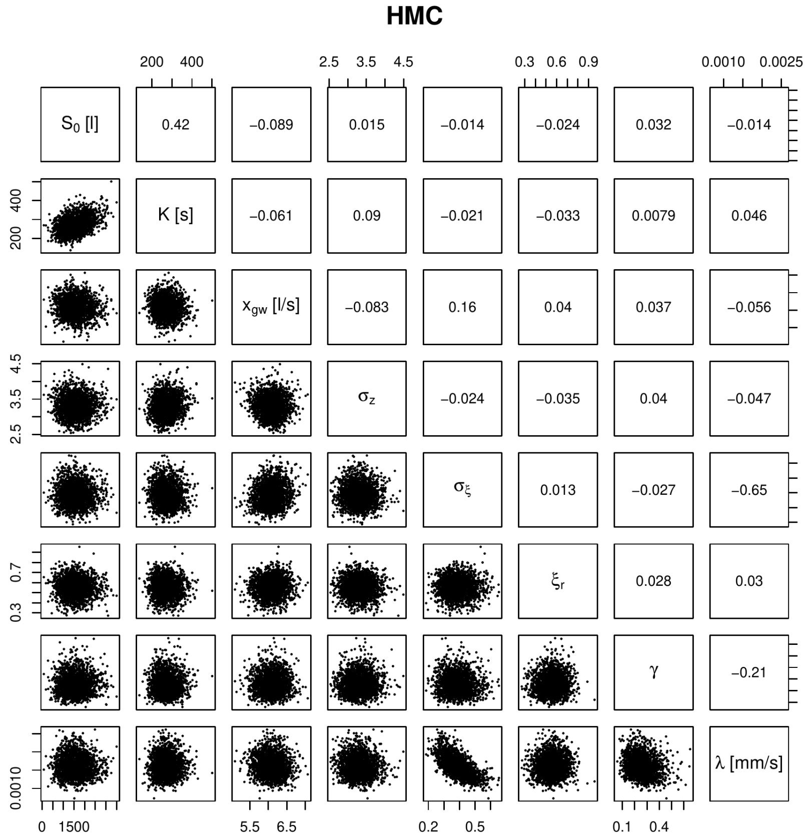


**Fig. S19** **2D marginal posteriors of parameters for HMC, scenario 1.**

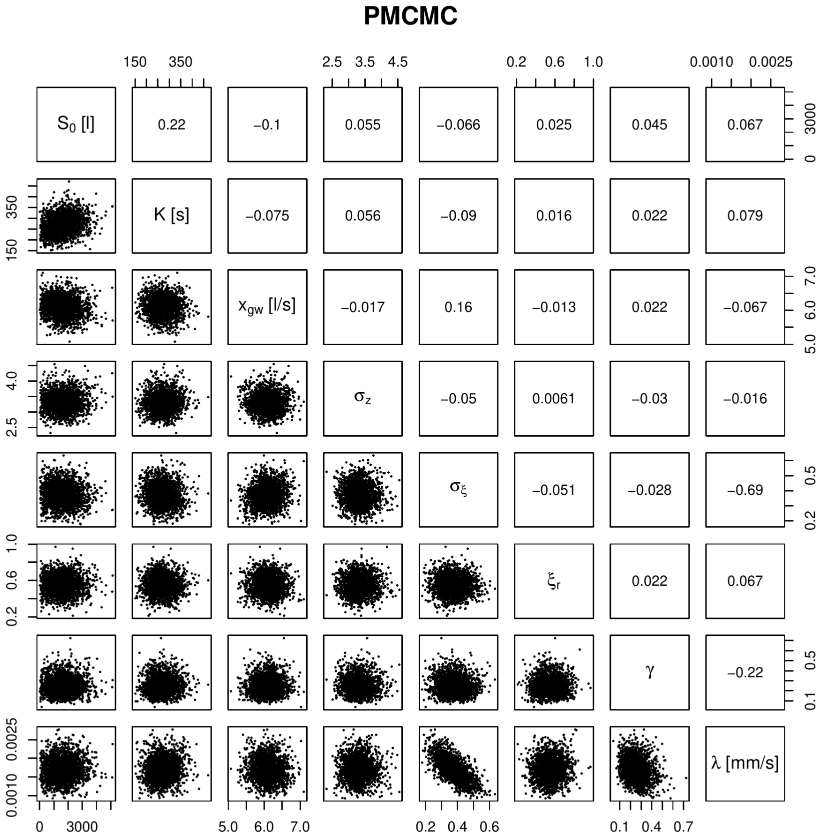


**Fig. S20** **2D marginal posteriors of parameters for PMCMC, scenario 1.**

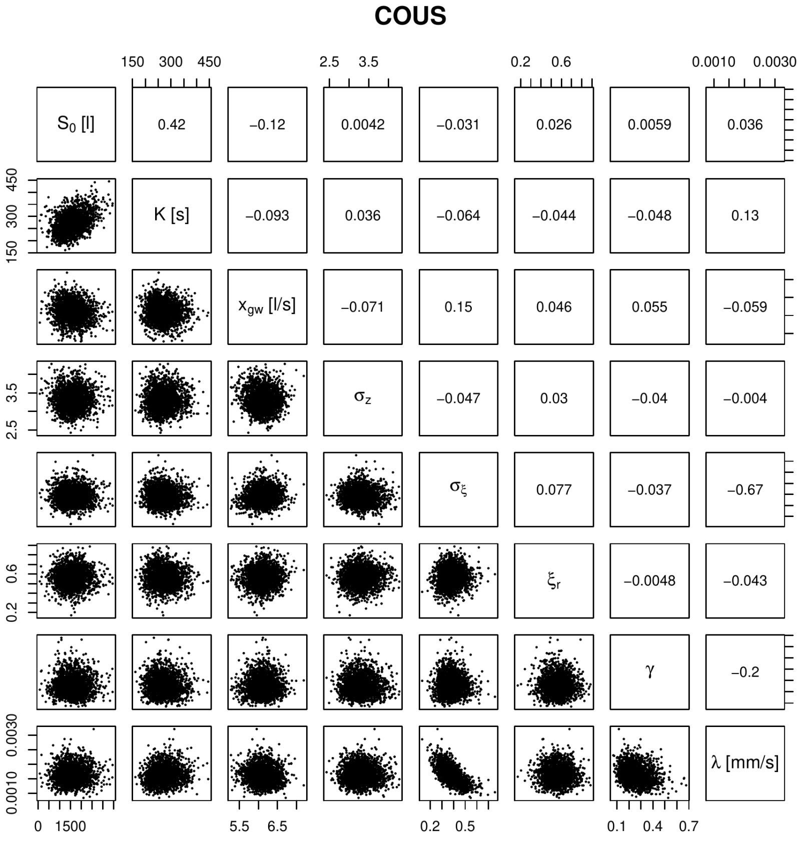


**Fig. S21** **2D marginal posteriors of parameters for COUS, scenario 1.**
